# Platelet PD-L1 reflects collective intratumoral PD-L1 expression and predicts immunotherapy response in non-small cell lung cancer

**DOI:** 10.1038/s41467-021-27303-7

**Published:** 2021-12-01

**Authors:** Clemens Hinterleitner, Jasmin Strähle, Elke Malenke, Martina Hinterleitner, Melanie Henning, Marco Seehawer, Tatjana Bilich, Jonas Heitmann, Martina Lutz, Sven Mattern, Sophia Scheuermann, Marius Horger, Stefanie Maurer, Juliane Walz, Falko Fend, Rupert Handgretinger, Christian Seitz, Bettina Weigelin, Stephan Singer, Helmut Salih, Oliver Borst, Hans-Georg Kopp, Lars Zender

**Affiliations:** 1 Department of Medical Oncology & Pneumology (Internal Medicine VIII), University Hospital Tuebingen, Tuebingen, Germany; 2 DFG Cluster of Excellence 2180 ‘Image-guided and Functional Instructed Tumor Therapy’ (iFIT), University of Tuebingen, Tuebingen, Germany; 3 Department of Hematology, Oncology and Immunology, University Hospital Tuebingen, Tuebingen, Germany; 4 Department of Pediatric Hematology and Oncology, University Hospital Tuebingen, Tuebingen, Germany; 5 Institute for Cell Biology, Department of Immunology, University of Tuebingen, Tuebingen, Germany; 6 Clinical Collaboration Unit Translational Immunology, German Cancer Consortium (DKTK), Department of Internal Medicine, University Hospital Tuebingen, Tuebingen, Germany; 7 Department of Pathology and Neuropathology, University Hospital Tuebingen, Tuebingen, Germany; 8 Department of Radiology, University Hospital Tuebingen, Tuebingen, Germany; 9 Department of Radiology, Memorial Sloan Kettering Cancer Center, New York, NY USA; 10 Werner Siemens Imaging Center, Department of Preclinical Imaging and Radiopharmacy, Eberhard Karls University Tuebingen, Tuebingen, Germany; 11 University Hospital, Department of Cardiology and Angiology, Eberhard Karls University of Tuebingen, Tuebingen, Germany; 12 Robert-Bosch-Hospital, Department of Molecular and Pneumological Oncology, Stuttgart, Germany; 13 German Cancer Research Consortium (DKTK), Partner Site Tübingen, German Cancer Research Center (DKFZ), Heidelberg, Germany

**Keywords:** Non-small-cell lung cancer, Tumour biomarkers, Tumour immunology

## Abstract

Immune-checkpoint inhibitors (ICI) have transformed oncological therapy. Up to 20% of all non-small cell lung cancers (NSCLCs) show durable responses upon treatment with ICI, however, robust markers to predict therapy response are missing. Here we show that blood platelets interact with lung cancer cells and that PD-L1 protein is transferred from tumor cells to platelets in a fibronectin 1, integrin α5β1 and GPIbα-dependent manner. Platelets from NSCLC patients are found to express PD-L1 and platelet PD-L1 possess the ability to inhibit CD4 and CD8 T-cells. An algorithm is developed to calculate the activation independent adjusted PD-L1 payload of platelets (pPD-L1^Adj.^), which is found to be superior in predicting the response towards ICI as compared to standard histological PD-L1 quantification on tumor biopsies. Our data suggest that platelet PD-L1 reflects the collective tumor PD-L1 expression, plays important roles in tumor immune evasion and overcomes limitations of histological quantification of often heterogeneous intratumoral PD-L1 expression.

## Abstract (web summary)

The definition of biomarkers to predict therapy responses to immune-checkpoint inhibitors represent an unmet clinical need. Here the authors provide evidences that platelet-derived PD-L1 could serve as a prognostic and predictive biomarker in patients with non-small cell lung cancer.

## Introduction

Immune-checkpoint receptors like CTLA4 and PD-1 are crucial for preventing excessive immune responses and autoimmunity^1,2^. Seminal discoveries made by Allison and Honjo provided preclinical proof of concept data that blockage of CTLA4 and PD1 signaling unleashes marked anti-tumor immune responses^3,4^. Clinical evaluation revealed remarkable therapeutic potential of immune-checkpoint inhibition in human cancer patients and for the first time allowed for long term survival of patients with advanced metastasized solid tumors^5–9^. Besides melanoma patients, especially patients suffering from non-small cell lung cancer (NSCLC) benefit from treatment with antibodies inhibiting the PD1 and CTLA4 immune checkpoints^5–7^. Nevertheless, simple and robust biomarkers to predict therapy responses towards ICI are still missing.

With 1.8 million deaths per year, lung cancer represents one of the most frequent and lethal cancers worldwide^10^. In the US 254,170 new lung cancer cases are expected to be diagnosed in 2021^11^. Given the high frequency of lung cancer and the cost of checkpoint inhibitory therapies, the lack of robust biomarkers to select patients who best possibly benefit from ICI represents a major burden for our health systems. Histological quantification of intratumoral PD-L1 expression is routinely performed in an attempt to predict therapy responses towards ICI, however, only an insufficient correlation between detection of PD-L1 expression in tumor biopsies and the overall response rate (ORR) was found^12^. In lung cancer, evaluation of smoking history, tumor mutational burden (TMB), microsatellite instability (MSI), high expression of CTLA4, low expression of CX3CL1 and infiltration of CD8+ T cells within the tumor microenvironment (TME) seems to be superior in predicting therapy responses towards anti-PD-1/PD-L1 directed ICI^13–15^ when compared to histopathological PD-L1 quantification, however these markers so far could not be translated into a robust and clinically easy to use biomarker signature.

Here, we show that platelets, during their frequent interaction with tumor cells, ingest PD-L1 and present it on their surface, a process which is dependent on fibronectin, α5β1 and GPIbα. PD-L1 expressing platelets are detected in the TME and peripheral blood of NSCLC patients. The functionality of platelet PD-L1 (pPD-L1) is confirmed by inhibition of CD4+ and CD8+ activity. pPD-L1 correlates with tumor stage/grade and the occurrence of metastases. We develop an algorithm allowing to calculate the total PD-L1 payload of platelets (pPD-L1^Adj.^) without the need of artifact prone in vitro stimulation procedures. Strikingly, in our study pPD-L1^Adj.^ is shown to be superior in predicting response to ICI when compared to immunohistochemistry-based quantification of PD-L1 on tumor biopsies.

## Results

### Tumor cells transfer PD-L1 to platelets

**Fig. 1:**
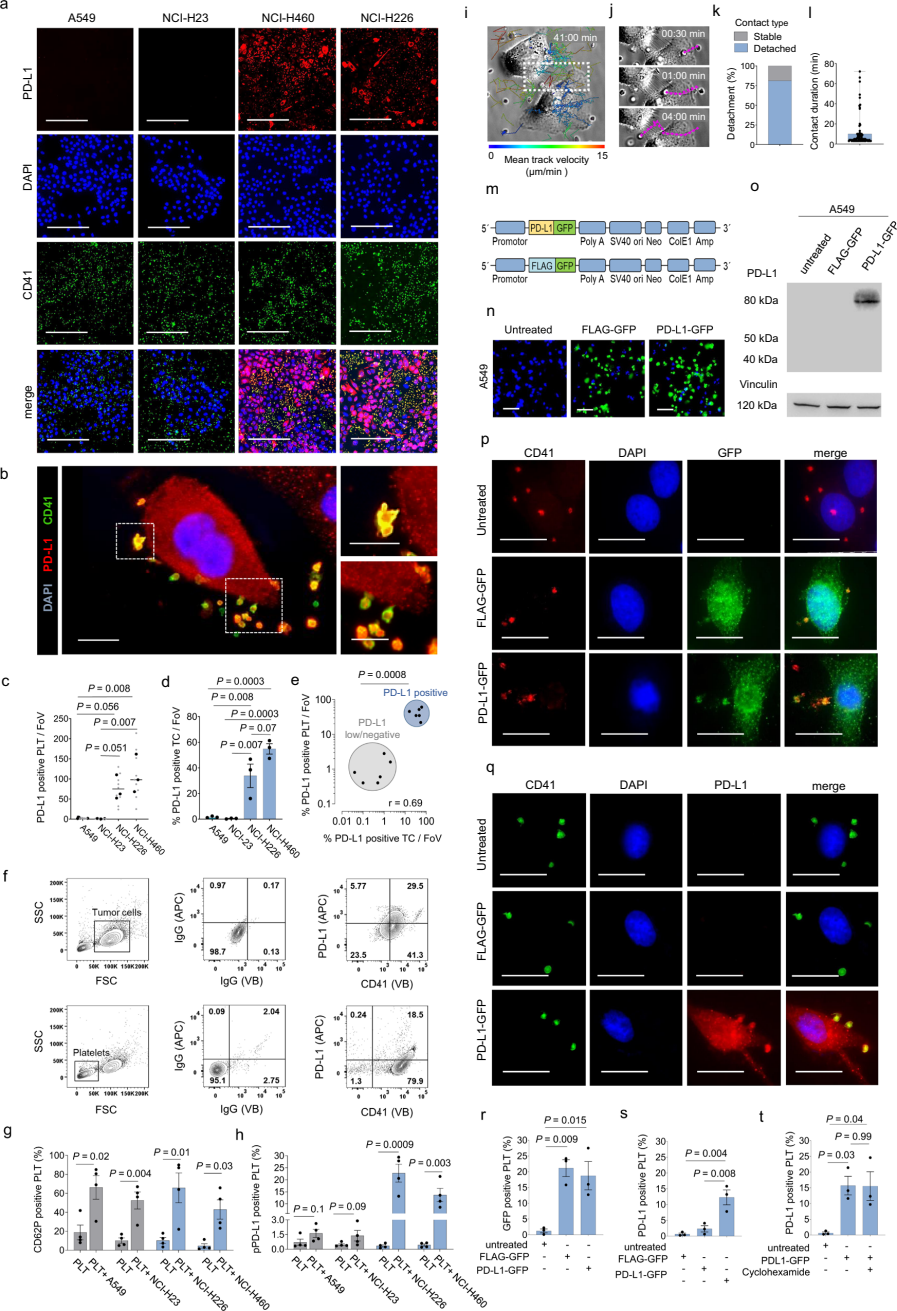
Direct platelet-tumor cell interactions result in PD-L1 expression on the platelet surface. **a** Representative immunofluorescence staining of PD-L1 (red)and the platelet marker CD41 (green) in four different NSCLC tumor cell lines (A549, NCI-H23, NCI-H460, NCI-H226) co-incubated with human platelets (*n* = 3 biological replicates). Scale bars 200 μm. **b** Immunofluorescence microscopy of NCI-H460 cells interacting with human platelets derived from a healthy donor (PD-L1: red, CD41: green) (*n* = 3). Scale bar left 20 µm, right 10 µm. **c** Quantitative analysis of the PD-L1+ platelets per field of view (FoV), analyzed by immunofluorescence microscopy (*n* = 9 FoV (small symbols) were analyzed out of a total of *n* = 3 independent experiments (large symbols)). Horizontal lines represent means. **d** Percentage of PD-L1+ tumor cells per FoV (*n* = 3). Data are mean ± SEM. **e** Correlation of % PD-L1+ platelets/FoV vs. % PD-L1 tumor cells/FoV (*n* = 3). Correlation was determined by simple linear regression analysis. **f** Flow cytometry gating strategy for the quantification of PD-L1+ tumor cells (upper) and platelets (lower) after co-incubation. **g**, **h** Surface expression of PD-L1 and CD62P on control platelets (PLT) and platelets after co-incubation with A549, NCI-H23, NCI-H226, NCI-H460 cells (*n* = 4). Data are mean ± SEM. Statistical significance was calculated by two-tailed Student’s *t* test. **i** Phase-contrast image of A549 tumor cells after 41 min coculture with platelets (ratio 1:1000). Overlaid migration tracks were color-coded based on their mean velocity. **j** Image sequence depicting tumor cell interaction and protrusion of a single platelet followed by detachment derived from zoom-in area indicated in (**i**). **k** Percentage of stable platelet–tumor cell contacts lasting from contact initiation until the end of the observation period (total observation time: 41 min). **l** Contact duration of platelet–tumor cell interactions. Data derived from the analysis of *n* = 75 platelets out of one independent experiment. Boxes represent median and 25th to 75th percentiles, whiskers are minimum to maximum. **m** Scheme of vectors expressing PD-L1-GFP and FLAG-GFP used for transfection. **n** Immunofluorescence stainings of GFP of A549 cells transfected with FLAG-GFP or PD-L1-GFP (*n* = 3). Scale bar 50 µm. **o** Western blot analysis for PD-L1 in untreated and transfected A549 cells (*n* = 2). Vinculin was used as loading control. Presentation of full scan blots are provided in the Source data file. **p**, **q** Representative immunofluorescence microscopy of untreated, FLAG-GFP and PD-L1-GFP-transfected A549 cells interacting with platelets (*n* = 3). Tumor cells and platelets were stained for GFP (upper) and PD-L1 (lower). Scale bar 50 µm. **r**, **s** Flow-cytometry-based quantification of GFP (l) and PD-L1 (**m**) on platelet surfaces after co-incubation with untreated and transfected A549 cells (*n* = 3). **t** Expression of PD-L1 on platelets pre-treated with 100 µM cycloheximide after co-incubation with PD-L1-GFP-transfected A549 cells (*n* = 3). **r**–**t** Data are mean ± SEM. **c**, **d, r**–**t** Statistical significance was calculated by one-way ANOVA and Tukey’s multiple comparisons test. Source data are provided as a Source Data file.

To address whether the immune regulatory protein PD-L1 can be transferred from tumor cells to platelets, we co-incubated platelets obtained from healthy donors with four different NSCLC tumor cell lines harboring varying expression levels of PD-L1 (NCI-H23, A549: PD-L1 low/negative, NCI-H226, NCI-H460: PD-L1 positive) (Fig. 1a, b). PD-L1 positivity was determined by flow cytometry and defined as PD-L1 expression in ≥ 5% of all tumor cells. PD-L1 expression on platelets (pPD-L1) was observed after co-incubation with the PD-L1 expressing NCI-H226 and NCI-H460 cells but not after co-incubation with the PD-L1 low/negative cell lines NCI-H23 and A549 (Fig. 1c–e). Results were validated using a flow cytometry-based approach (Fig. 1f). Co-incubation of platelets with all tumor cell lines resulted in platelet activation, as indicated by P-selectin (CD62P) induction (Fig. 1g), however only co-incubation with PD-L1 positive NCI-H226 and NCI-H460 cells resulted in an increased PD-L1 expression on the platelet surface (Fig. 1h). To ensure that platelets from healthy donors, used in this assay, do not harbor relevant amounts of endogenous PD-L1 we conducted western blot analyses on platelet whole-cell lysates. Indeed, Western Blot data confirmed that platelets from healthy donors do not express relevant PD-L1 levels (Supplementary Fig. 5b).

Of note, conditioned medium from tumor cells induced platelet activation but did not result in increased levels of PD-L1 protein on the platelet surface (Supplementary Fig. 3c, d), suggesting that PD-L1 transfer from tumor cells to platelets is dependent on a direct cell-cell contact between both cell types. Of note, frequent interaction with platelets was not restricted to adherent tumor cells but could for example also be observed for non-adherent A549 lung cancer cells (Supplementary Fig. 3e, f).

To gain deeper insights into the interaction of platelets and lung cancer cells, we took advantage of a live-cell imaging platform, where platelets are added to the medium and circulate through an imaging chamber that contains human NSCLC cells. Real time video microscopy revealed distinct interactions of tumor cells and platelets (Fig. 1i, j and Supplementary Movie 1). Strikingly, platelets remained fully agile and re-entered the circulation after contacting the tumor cell membrane (Fig. 1k–j and Supplementary Movies 1, 2). These data suggest that platelets can re-circulate after tumor cell attachment and activation and are in line with studies by Cloutier and Michaelson et al.^16,17^.

While platelets are anuclear, protein translation from RNA can nevertheless occur within platelets^16–18^. We therefore set out to investigate whether PD-L1 expression in platelets depends on a transfer of PD-L1 protein from tumor cells to platelets or whether a transfer of PD-L1 mRNA with subsequent protein synthesis within the platelet is involved. Transfection of vectors encoding for PD-L1-GFP and FLAG-GFP fusion proteins into PD-L1 negative A549 cells (Fig. 1m–o) resulted in high numbers of GFP positive platelets upon co-incubation (Fig. 1p–s). As inhibition of protein translation in platelets by cycloheximide did not result in a reduction of PD-L1-GFP expression in platelets (Fig. 1t), our data suggest that PD-L1 protein transfer and not mRNA transfer is underlying the observed pPD-L1 expression after interaction of platelets and tumor cells^19,20^.

**Fig. 2:**
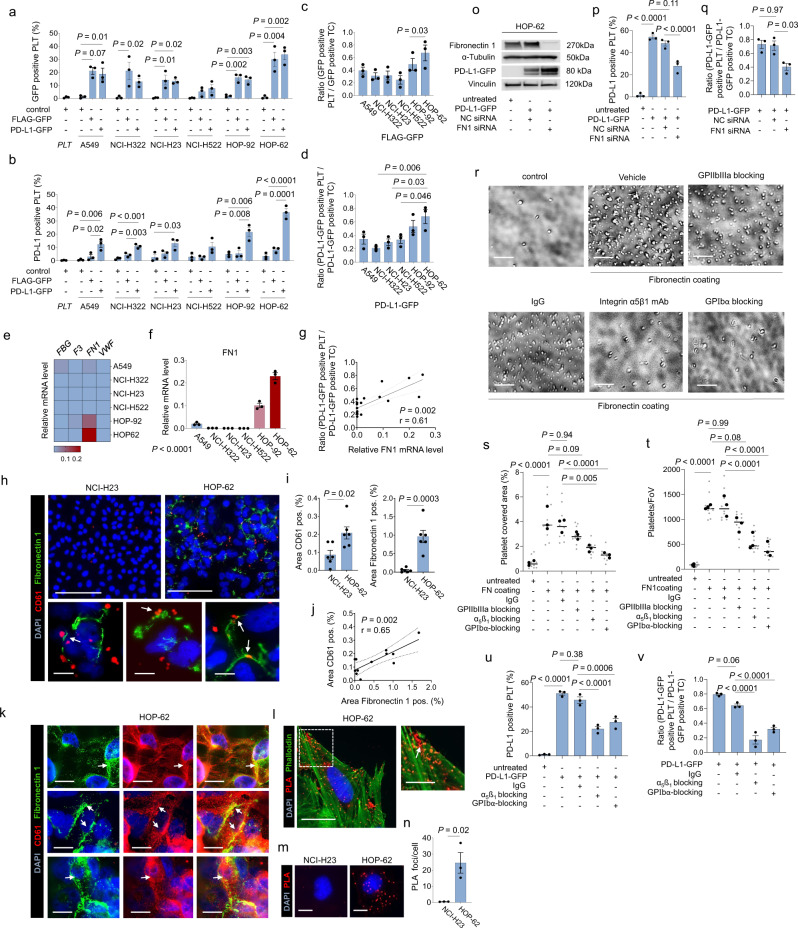
Fibronectin mediates platelet adhesion to tumor cells and facilitates PD-L1 protein transfer. **a**, **b** Expression of GFP (**a**) and PD-L1 (**b**) on platelet surfaces after co-incubation with six FLAG-GFP- and PD-L1-GFP--transfected NSCLC cell lines (A549, NCI-H322, NCI-H522, NCI-H23, HOP-62, HOP-92) (*n* = 3). **c**, **d** Ratio of GFP+ platelets/GFP+ tumor cells (**c**) and PD-L1-GFP+ platelets/PD-L1-GFP+ tumor cells (**d**) after co-incubation of platelets with transfected NSCLC cell lines (*n* = 3). **e** Heat map of relative fibrinogen (*FBG*), tissue factor (*F3*), fibronectin 1 (*FN1*) and von Willebrand factor (*VWF*) mRNA levels in all tested NSCLC cell lines (*n* = 3). **f** Relative mRNA level of *FN1* in all tested NSCLC cell lines (*n* = 3). **g** Correlation ratio of PD-L1-GFP+ platelets/PD-L1-GFP+ tumor cells and relative *FN1* mRNA level (*n* = 3). **h** Immunofluorescence images of platelet adhesion to NCI-H23 (upper left) and HOP-62 cells (upper right and lower) (fibronectin: green, platelets: red) (*n* = 3). Upper scale bar 100 µm, lower 20 µm. **i** Left, Quantification of adhesive platelets after co-incubation with NCI-H23 and HOP-62. Quantified as CD61+ area in %/FoV (*n* = 6 out of 3 independent experiments). Right, quantification of fibronectin covered area in %/FoV in NCI-H23 and HOP-62 cells (*n* = 6 out of 3 independent experiments). **j** Correlation of platelet and fibronectin covered area in %/FoV. **k** Immunofluorescence images of PD-L1 and fibronectin expression in HOP-62 cells (*n* = 2). Scale bar 20 µm. **l** Representative PLA with PD-L1 and fibronectin in HOP-62 cells. Scale bar left 20 µm, right 10 µm (*n* = 1). **m** Representative PLA with PD-L1 and fibronectin in NCI-H23 and HOP-62 cell (*n* = 3). Scale bar 10 µm. **n** PLA quantification of foci/cell in 119 NCI-H23 and 126 HOP-62 cells out of three biological replicates. **o** Western blot for PD-L1 and fibronectin in PD-L1-GFP-transfected HOP-62 cells after siRNA knockdown for fibronectin. Vinculin and α-Tubulin were used as loading controls (*n* = 2). Presentation of full scan blots are provided in the Source data file. **p** Expression of PD-L1 on platelets after co-incubation with PD-L1-GFP, PD-L1-GFP/siFN1, and PD-L1-GFP/siNC-transfected HOP-62 cells (*n* = 3). **q** Ratio PD-L1-GFP+ platelets/PD-L1-GFP+ tumor cells after co-incubation of platelets with PD-L1-GFP, PD-L1-GFP/siFN1, and PD-L1-GFP/siNC-transfected HOP-62 cells (*n* = 3). **r** Representative images of platelet adhesion to fibronectin-coated surface in the presence or absence of different platelet-blocking agents (*n* = 3). Scale bar 20 µm. **s**, **t** Quantitative analysis of the platelet adhesion assay as platelet covered area/FoV in % (s) and platelets/FoV (t) (*n* = 9 (small symbols) were analyzed out of three independent experiments (large symbols)). Horizontal lines represent mean. Statistical significance was calculated by one-way ANOVA and Tukey’s multiple comparisons test. **u** Quantification of PD-L1 on platelets after co-incubation with PD-L1-GFP-transfected HOP-62 cells with or without pre-treatment with platelet-blocking agents (*n* = 3). **v** Ratio PD-L1-GFP+ platelets/PD-L1-GFP+ tumor cells after co-incubation of platelets with PD-L1-GFP-transfected HOP-62 cells with or without pre-treatment with platelet-blocking agents (*n* = 3). **a**–**d**, **f**, **p**, **q**, **u**, **v** Data are mean ± SEM. Statistical significance was calculated by one-way ANOVA and Tukey’s multiple comparisons test. **g, j** Correlation was determined by simple linear regression analysis. **I, n** Data are mean ± SEM. Statistical significance was calculated by two-tailed Student’s *t* test. Source data are provided as a Source Data file.

While the transfer of PD-L1-GFP or FLAG-GFP was robustly observed across various NSCLC cell lines, we nevertheless noted differences in protein transfer efficacies. For example, platelets showed low levels of FLAG-GFP and PD-L1-GFP after co-incubation with NCI-H322, NCI-H522 and NCI-H23 cells, while HOP-62 and HOP-92 cells displayed significantly higher protein transfer rates (Fig. 2a–d). Given that our data indicated that a platelet-tumor-cell contact is necessary for a sufficient transfer of PD-L1 from tumor cells to platelets, we hypothesized that expression levels of adhesion molecules might determine the efficacy of protein transfer from tumor cells to platelets. Along these lines we found that PD-L1 transfer rates positively correlated with fibronectin (FN1) mRNA expression levels, while no significant correlation was found for fibrinogen alpha chain (FGA) or tissue factor (F3) mRNA expression (Fig. 2e–g). Of note, fibronectin expression also correlated with platelet-tumor cell interaction in vitro (Fig. 2h–j). Immunofluorescence staining as well as analysis of protein–protein interaction via proximity ligation assay (PLA) revealed close proximity of fibronectin and PD-L1 (Fig. 2k–n) at the cell surface. In line with these observations, we found that siRNA mediated knockdown of fibronectin resulted in a significant reduction of PD-L1 transfer, thus functionally validating fibronectin as a key regulator of protein transfer from tumor cells to platelets (Fig. 2o–q).

Platelet adhesion to fibronectin is known to be mediated via several molecules including GPIbα, integrin α_5_ß_1_ or GPIIbIIIa^21,22^. We therefore set out to address whether inhibition of these adhesion molecules on platelets reduces adhesion to fibronectin and PD-L1 uptake from tumor cells. Strikingly, while monoclonal antibodies against GPIbα and integrin α_5_ß_1_ prevented platelet adhesion to fibronectin (Fig. 2r–t) and PD-L1 protein transfer (Fig. 2u, v), inhibition of GPIIbIIIa by Tirofiban only marginally reduced platelet adhesion (Fig. 2r–t).

### Detection of functional PD-L1 on platelets of NSCLC patients

**Fig. 3:**
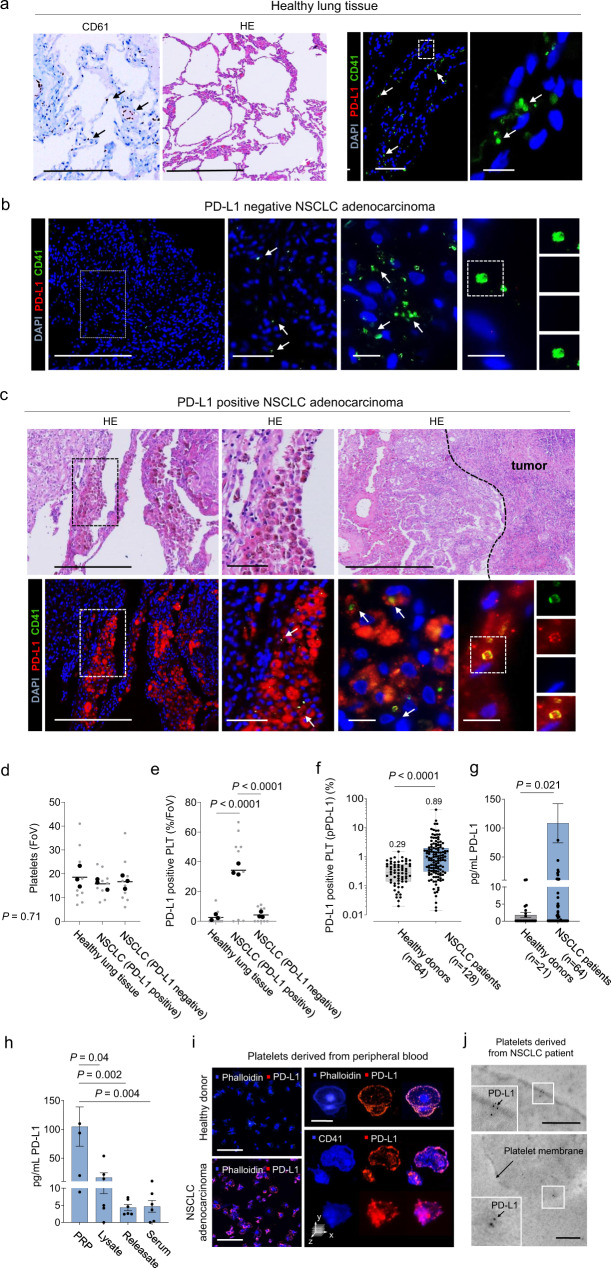
Platelets from NSCLC patients show increased PD-L1 levels. **a** Left panel, left, Immunohistochemistry for CD61 in healthy human lung tissue (black arrow highlights CD61+ platelets). Scale bar 200 µm. Left panel, right, Representative micrograph of healthy lung tissue (H&E). Scale bar 500 µm. Right panel, Immunofluorescence microscopy for CD41+ (green), PD-L1- (red) platelets in healthy lung tissue (*n* = 3). Scale bar left 100 µm, right 10 µm. **b** Immunofluorescence staining for CD41 (green) and PD-L1 (red) on platelets in a PD-L1− NSCLC patient tumor sample (*n* = 3). Scale bar left 500 µm, center left 50 µm, center right and right 10 µm. **c** Upper, Representative micrographs of NSCLC adenocarcinoma (H&E) (*n* = 3). Scale bar left 250 µm, center 50 µm, right 500 µm. Lower, Immunofluorescence staining for CD41 (green) and PD-L1 (red) (*n* = 3). Scale bar left 500 µm, center left 50 µm, center right and right 10 µm. **d**, **e** Quantitative analysis of the platelets/FoV (d) and PD-L1+ platelets (%/FoV) (**e**) in healthy lung tissue, PD-L1+ and PD-L1- NSCLC patients (*n* = 9) (small symbols) were analyzed out of three independent experiments in three healthy donors and six NSCLC patients (large symbols). Horizontal line represent mean. **f** Percentage of PD-L1+ platelets in healthy donors (*n* = 64) and NSCLC patients (*n* = 128). Each dot represents a single donor. Boxes represent median and 25th to 75th percentiles, whiskers are minimum to maximum. **g** Total amount of PD-L1 (pg/mL) in healthy donors (*n* = 21) and NSCLC patients (*n* = 64) analyzed by ELISA. Protein level were analyzed in 64 out of 128, randomly assigned patients of the NSCLC cohort. Data are mean ± SEM. **h** Total amount of PD-L1 (pg/mL) in PRP, platelet lysate, platelet releasate, and serum (*n* = 6). Data are mean ± SEM **i** Left, Representative PD-L1 immunofluorescence staining of platelets from a healthy donor (upper) and a NSCLC patient (lower). Platelets were counter stained with phalloidin. Right, Expression pattern of PD-L1 on a platelet derived from a NSCLC patient counter stained with phalloidin (upper) or CD41 (lower). Scale bar left 10 µm, right 2 µm (*n* = 1). **j** Platelets of a NSCLC patient, assessed by transmission electron microscopy. PD-L1 stained with post-embedding immunogold-labeling. PD-L1 gold particles densely accumulating on the platelet membrane (black dots). Upper scale bar 2 µm, lower 100 nm (*n* = 1). **d**, **e, h** Statistical significance was calculated by one-way ANOVA and Tukey’s multiple comparisons test. **f**, **g** Statistical significance was calculated by Mann–Whitney test. Source data are provided as a Source Data file.

To address the significance of our findings for human cancers, we next quantified PD-L1 expression on platelets in healthy lung tissue or NSCLC tumor tissue. While platelets were detected in high abundance in healthy lung tissue and PD-L1 negative NSCLC, we could not observe any relevant PD-L1 expression on these platelets (Fig. 3a, b, d, e). In contrast PD-L1 positive platelets were observed in high abundance in tissue sections from patients suffering from PD-L1 positive NSCLC (Fig. 3c–e). To quantify the number of PD-L1 positive platelets outside the tumor, we next isolated platelets from the peripheral blood of a cohort of 64 healthy donors and 128 NSCLC patients. Fluorescence-Activated Cell Sorting (FACS) revealed threefold higher numbers of PD-L1 positive platelets in NSCLC patients as compared to healthy donors (median pPD-L1 expression in healthy donors 0.29 (95%CI: 0.21 – 0.44), median pPD-L1 expression in NSCLC patients 0.89 (95%CI: 0.61–1.21) (Fig. 3 f). The detected differences were even higher, when total pPD-L1 levels were determined using a quantitative enzyme-linked immunosorbent assay (ELISA). While platelet rich plasma (PRP) from NSCLC patients in average contained 108.3 pg/mL PD-L1, PRP from healthy volunteers only contained 1.8 pg/mL (Fig. 3g, h). Differences in pPD-L1 expression in NSCLC patients versus healthy volunteers were also confirmed using western blot analysis (Supplementary Fig. 5b–d). Interestingly, PD-L1 expression was highest in patients with advanced (UICC stage IV) tumors (Supplementary Fig. 5e). Of note, immunofluorescence (Fig. 3i) and immunoelectron microscopy (Fig. 3j) revealed frequent PD-L1 clusters in platelets obtained from peripheral blood of a PD-L1 positive NSCLC patient, further underlining functionality of pPD-L1, as immune ligand clustering has been described to be a prerequisite for proper binding to its receptor^23^.

**Fig. 4:**
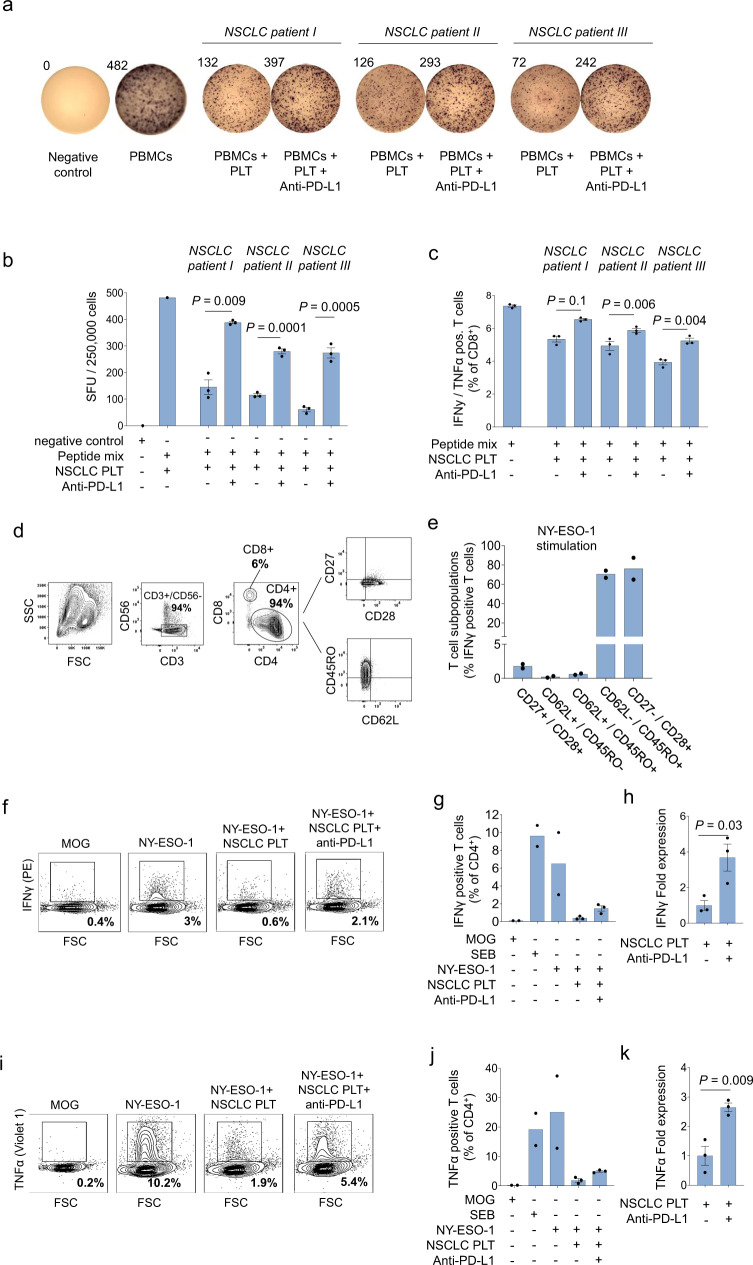
PD-L1 on platelets shows functional relevance via decreasing T-cell activity. **a** IFNγ ELISPOT assay of peptide-specific T-cells co-incubated with PD-L1+ platelets with or without anti-PD-L1 mAb pre-treatment (*n* = 3). **b** Quantification of the IFNγ ELISPOT assays (*n* = 3). **c** Flow cytometry-based quantification of indicated cytokines and surface markers for peptide stimulated CD8+ T-cells co-incubated with PD-L1+ platelets with or without anti-PD-L1 mAb pre-treatment (*n* = 3). **d** Representative FACS plots showing the gating strategy and the T-cell subpopulations after pre-sensitization, enrichment, and expansion. **e** Quantitative sub-phenotyping of NY-ESO-1 specific T cells using flow cytometry (*n* = 2). **f** Representative FACS plots displaying CD4+ TEM activity levels measured by INFγ expression after co-incubation with PD-L1+ platelets with or without anti-PD-L1 mAb pre-treatment (*n* = 3). **g** Quantification of INFγ+CD4+ TEM (*n* = 3). **h** IFNy fold change in CD4+ TEM (*n* = 3). **i** Representative FACS plots displaying CD4+ TEM activity levels measured by TNFα expression after co-incubation with PD-L1+ platelets with or without anti-PD-L1 mAb pre-treatment (*n* = 3). **j** Quantification of TNFα+CD4+ TEM (*n* = 3). **k**, TNFα fold change in CD4+ TEM (*n* = 3). **b**, **c**, **g**, **h**, **j**, **k** Data are mean ± SEM. Statistical significance was calculated by two-tailed Student’s *t* test. Source data are provided as a Source Data file.

Prompted by these results, we next explored whether pPD-L1 exerts immune-inhibitory functions. We stimulated human T cells from healthy donors with EBV/CMV-derived peptides in the presence or absence of PD-L1 positive platelets obtained from NSCLC patients. T cell activation was evaluated using an enzyme-linked-immuno-Spot (ELISpot) assays determining the effector cytokines IFNy and TNFα. In line with published data we observed that platelets dampen T cell activity independent of their PD-L1 expression status (Fig. 4a–c and Supplementary Fig. 6a, b)^24,25^. However, when PD-L1 expressing platelets were pre-treated with the anti-PD-L1 mAb Atezolizumab their T cell inhibitory effect was abolished (Fig. 4a–c). Next, we expanded our work towards tumor-associated antigens. New York esophageal squamous cell carcinoma 1 antigen (NY-ESO-1) belongs to the family of cancer-testis antigens, but is also aberrantly expressed in many tumor entities including NSCLC^26^. Stimulation of T cells from healthy donors with NY-ESO-1 peptides predominantly resulted in a clonal expansion of NY-ESO-1 specific CD4^+^ T cells (CD62L−/CD45RO + and CD27−/CD28+) (Fig. 4d), which were further specified as CD4+ effector memory T cells (T_EM_, CD62L−/CD45RO+ and CD27−/CD28+) (Fig. 4d, e). Remarkably, T_EM_ activity, as determined by IFNγ and TNFα release, decreased significantly upon co-incubation with PD-L1 positive platelets. However, T cell activity could be restored when pPD-L1 positive platelets were pre-treated with anti-PD-L1 (Fig. 4f–k).

**Fig. 5:**
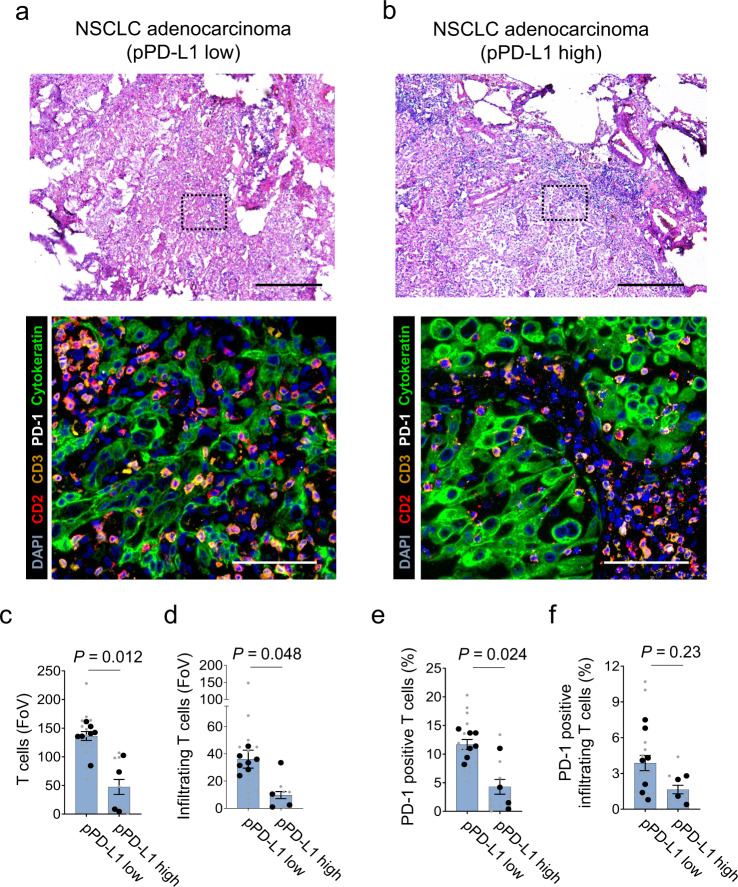
pPD-L1 correlates with T cell infiltration in NSCLC. **a**, **b** Upper, Representative micrographs of NSCLC adenocarcinoma (H&E) (*n* = 11). Scale bar 500 µm. Lower, Immunofluorescence staining for T-cells (CD2: red, CD3: orange and PD-1: white) in the TME of a NSCLC patient presenting with low pPD-L1 (**a**) and high pPD-L1 (**b**). Each image is representative for at least two regions of interest (ROI) in each tumor sample. ROI were selected based on manual prestaining of DAPI. Scale bar 100 µm (*n* = 11). **c**–**f** Quantification of T cells per FoV (**c**), PD-1+ T cells per FoV (%) (**d**), infiltrating T cells per FoV (**e**) and PD-1+ infiltrating T cells per FoV (%) (**f**). pPD-L1 high vs. low was defined according to the median expression in this cohort. A total number of *n* = 22 ROIs (small symbols) were analyzed out of a total of *n* = 11 patients (large symbols). Data are mean ± SEM. Statistical significance was calculated by two-tailed Student’s *t* test (**d**–**f**) or two-tailed Mann–Whitney test (**c**). Source data are provided as a Source Data file.

To investigate a potential impact of PD-L1 positive platelets on other immune cells, we also characterized changes in the overall immune cell composition (peripheral blood) in 10 NSCLC patients and five healthy controls (Supplementary Fig. 7a). In NSCLC patients pPD-L1 tended to be correlated negatively with the total number of NK (*p* **=** 0.1), CD4+ T cells (*p* **=** 0.09) and CD8+ T cells (*p* **=** 0.02) (Supplementary Fig. 7b). Moreover, in NSCLC patients more PD-1 and PD-L1 was expressed on dendritic cells (DCs), natural killer (NK) cells and CD4+ and CD8+ T cells (Supplementary Fig. 7c, d). In our analyses we did not observe a correlation of PD-1 or PD-L1 expression and pPD-L1 in DCs, NK cells or CD4+ T cells (Supplementary Fig. 7e, f). However, we detected a positive correlation of pPD-L1 and PD-1 on CD8+ T cells (*p* **=** 0.02). We also quantified T cells and T cell infiltration in the TME in eleven NSCLC patients with different levels of pPD-L1 using the MACSima ultradeep tissue profiling platform. Noteworthy, in patients with high pPD-L1 we observed lower numbers of T cells in the TME and less infiltrating T cells (Fig. 5a–d). In contrast to our findings in the peripheral blood, we observed an inverse correlation of PD-1 on T cells and pPD-L1 (Fig. 5e, f).

### Regulation of pPD-L1 during platelet activation

**Fig. 6:**
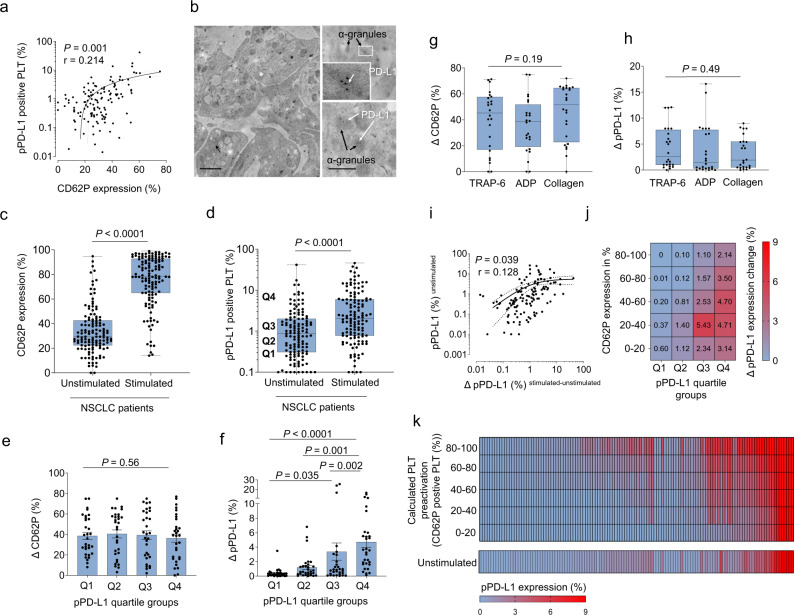
Platelets from NSCLC patients show increased PD-L1 protein levels upon activation. **a** Correlation between platelet-derived PD-L1 (pPD-L1) and platelet activation (CD62P expression) in 128 NSCLC patients. Each dot represents a single patient. **b** Platelets from a NSCLC patient assessed by transmission electron microscopy. PD-L1 stained with post-embedding immunogold-labeling. Upper and lower right, PD-L1 gold particles densely accumulate in α-granules. Scale bar left 1 µm, right 100 nm (*n* = 1). **c**, **d** Changes in CD62P (**c**) and the pPD-L1 (**d**) levels upon platelet stimulation with 10 µM TRAP-6 for 2 min in NSCLC patient samples (*n* = 128). Boxes represent median and 25th to 75th percentiles, whiskers are minimum to maximum. Statistical significance was calculated by two-tailed Mann–Whitney test. **e** CD62 expression change (ΔCD62P) in the different pPD-L1 quartile groups identified in unstimulated platelets of NSCLC patients (*n* = 128). Data are mean ± SEM. Statistical significance was calculated using Kruskal–Wallis test. **f** pPD-L1 expression change (ΔpPD-L1) in the different pPD-L1 quartile groups identified in unstimulated platelets of NSCLC patients (*n* = 128). Data are mean ± SEM. Statistical significance was calculated by Friedman and Dunn’s multiple comparisons test. **g** CD62P expression change (ΔCD62P, CD62P in stimulated platelets in % – CD62P in unstimulated platelets in %) after platelet activation with 10 µM TRAP-6 (*n* = 24), 2.5 µM ADP (*n* = 24), or 5 µg/ml collagen (*n* = 24). Boxes represent median and 25th to 75th percentiles, whiskers are minimum to maximum. **h** pPD-L1 expression change ΔpPD-L1 (pPD-L1 in stimulated platelets in % – pPD-L1 in unstimulated platelets in %) in NSCLC patients after platelet activation. Statistical significance was calculated by Kruskal–Wallis test. Boxes represent median and 25th to 75th percentiles, whiskers are minimum to maximum. **i** Correlation between pPD-L1 expression in unstimulated platelets of 128 NSCLC patients and the ΔpPD-L1 upon platelet stimulation with 10 µM TRAP-6. Each dot represents a single patient. **j** Heatmap showing the calculated ΔpPD-L1 depending on the CD62P activation ranges (*y*-axis) and the quartiles of pPD-L1 level (*x*-axis) calculated in pooled data from 128 NSCLC patients. For details of subsampling and calculation used, see Methods and Supplementary Fig. 4. **k** Adjusted pPD-L1 levels in all 128 NSCLC patients upon calculated platelet pre-activation ranges (by CD62P expression level). **a**, **i** Correlation was determined by simple linear regression analysis. Source data are provided as a Source Data file.

As it is well established that expression levels of platelet surface proteins correlate with the platelet activation status, we reasoned that different degrees of platelet activation might underlie varying levels of pPD-L1 expression on the platelet surface. Indeed, when we analyzed the platelet activation marker CD62P, we observed varying CD62P expression levels which showed a strong positive correlation with pPD-L1 expression (Fig. 6a). Of note, while PD-L1 expression in general was lower in unstimulated platelets, we were able to robustly detect pPD-L1 on the platelet surface of resting (CD62P negative) platelets (Supplementary Fig. 5a). In line with this, we also detected pPD-L1 in α-granules (Fig. 6b).

As even highly standardized blood collection procedures can result in varying levels of shear-stress mediated platelet activation and therefore complicates standardization^27,28^, we hypothesized that different levels of platelet pre-activation might complicate the interpretability and comparability of pPD-L1 levels on freshly collected platelets from different patients. We therefore reasoned that a controlled in vitro activation of platelets with subsequent maximization of pPD-L1 expression might most adequately uncover the total payload of platelet PD-L1 and best possibly allow a comparison between different patients. Indeed, we found that pPD-L1 expression was maximized upon controlled platelet stimulation with the PAR1 agonist TRAP-6 (Fig. 6c–f and Supplementary Fig. 8 a-d) or other platelet activation agents such as ADP or collagen (Fig. 6g, h) and thus might allow for a better comparability of pPD-L1 levels between different patients. However, controlled platelet activation and subsequent measurement of CD62P and pPD-L1 is technically demanding and might prevent the use of pPD-L1 as a biomarker in clinical routine. We therefore set out to explore whether a normalized PD-L1 level on the platelet surface could be calculated without in vitro manipulation of platelets. To do so we developed an adjustment model based on the calculation of ΔPD-L1 (ratio of PD-L1 before and after stimulation) as a function of pPD-L1 expression in unstimulated platelets and the degree of pre-activation (CD62P expression) (Fig. 6i, j). Specifically, we devised a matrix which allows us to calculate PD-L1^Adj.^ for subgroups of patients harboring different levels of platelet pre-activation (CD62P expression) and pPD-L1 expression (Fig. 6j and Supplementary Fig. 9). Taking advantage of our matrix, corrected PD-L1 levels, designated pPD-L1^Adj.^, were determined for all patients (Fig. 6k).

### Adjusted platelet-derived PD-L1 serves as a prognostic and predictive marker in NSCLC

**Fig. 7:**
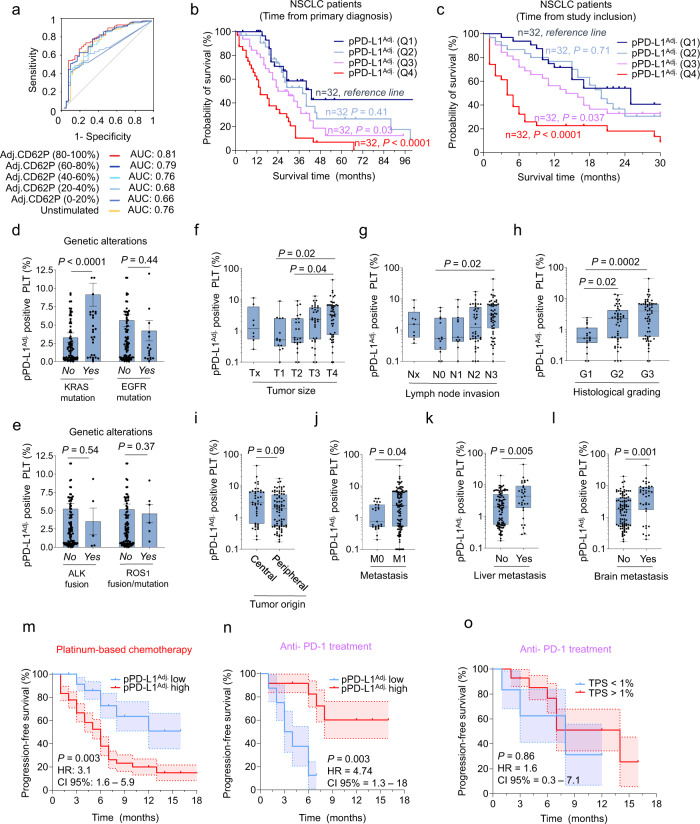
Platelet-derived PD-L1 (pPD-L1) as prognostic and predictive marker in NSCLC. **a** Combined estimate of platelet CD62P (0–100%) and pPD-L1 levels predicts overall survival (OS) in 128 NSCLC patients; receiver-operating characteristics (ROC) analysis. **b** Kaplan–Meier analysis of overall survival (OS) dependent on the pPD-L1^Adj^ as defined by quartile groups (very low (Q1), low (Q2), high (Q3) and very high (Q4)). Survival data refer to the time point of primary diagnosis (*n* = 128). **c** Overall survival (OS) in different pPD-L1^Adj^ quartile groups according to the time point of platelet analysis (*n* = 128). **b**, **c** Statistical significance was calculated by log-rank test. **d**, **e** Association of pPD-L1^Adj^ levels and different genetic alterations (KRAS (mut = 38, wt = 90), EGFR (mut = 19, wt =109), EML-4-ALK (alteration = 5, no alteration = 123) and ROS-1 (alteration = 6, no alteration 122)) (wt = wildtype, mut = mutation). Data are mean ± SEM. Statistical significance was calculated by two-tailed Mann–Whitney test. **f**–**h** pPD-L1^Adj^ levels in patients with different tumor stage ((f), Tx = 8, T1 = 14, T2 = 23, T3 = 33, T4 = 50), lymph node invasion ((**g**), Nx = 9, N0 = 13, N1 = 11, N2 = 40, N3 = 55), and grade ((**h**), G1 = 19, G2 = 48, G3 = 61). Each dot represents a single patient. Statistical significance was calculated by Kruskal–Wallis test and Dunn’s multiple comparisons test. **i** Association of pPD-L1^Adj^ levels with tumor origin (central = 42, peripheral = 79). **j**–**l** pPD-L1^Adj^ is associated with the occurrence of metastasis (in general ((**j**), M0 = 25, M1 = 103) and at specific sites, including liver (confirmed = 31, no metastasis = 94) and brain (confirmed = 37, no metastasis = 91). **f**–**l** Each dot represents a single patient. Boxes represent median and 25th to 75th percentiles, whiskers are minimum to maximum. Statistical significance was calculated by two-tailed Mann–Whitney test. **m** Kaplan–Meier curves estimates of PFS in patients with a pPD-L1^Adj^ level > median (red) and pPD-L1^Adj^ level < median (blue) treated with conventional chemotherapy (*n* = 62). **n** Kaplan–Meier curves estimates of PFS in patients with a pPD-L1^Adj^ level > median (red) and pPD-L1^Adj^ level < median (blue) treated with anti-PD-1 therapy (*n* = 20). **o** Kaplan–Meier curves estimates of PFS in patients with a TPS score > 1% (red) and TPS < 1% (blue) treated with anti-PD-1 therapy (*n* = 20). **m**–**o** Statistical significance was calculated by log-rank test. Source data are provided as a Source Data file.

We first used the calculated pPD-L1^Adj.^ levels and performed a receiver-operating characteristic (ROC) analysis for overall survival (OS). We found that pPD-L1^Adj.^ levels in the subgroup of maximal platelet activation (CD62P 80–100%) showed highest accuracy in predicting OS and were superior in predicting OS compared to pPD-L1 (Fig. 7a). Since no cut-off value for pPD-L1^Adj^ had been established so far, we analyzed OS in pPD-L1^Adj.^ quartile groups (Q1-4) using the Kaplan‐Meier method (Fig. 7b, c). Details regarding characteristics of our patient population are provided in Supplementary Table 1. The median observation time for monitoring OS in our study was 23.5 months (95%CI: 3.4–67.55 months). At data cutoff for overall survival, 42 of 128 patients (32.8%) were still alive. Strikingly, patients with high pPD-L1^Adj.^ levels showed a significantly shortened OS (Fig. 7b). The median survival in Q1 (low pPD-L1^Adj.^ levels) was 43 months compared to only 24 months in Q3 (high pPD-L1^Adj.^ levels) (hazard ratio (HR) for death Q1 vs. Q3: 2 (95%CI: 1–3.9)) and 14 months in Q4 (very high pPD-L1^Adj.^ levels) (hazard ratio (HR) for death Q1 vs. Q4: 3.64 (95%CI: 1.97–6.72)). Importantly, the observed differences in OS were not restricted to the time since initial diagnosis but were still significant when analyzing the time period since platelet analysis (Fig. 7c).

It has been reported that mutations in key oncogenic drivers do not only fuel proliferation via cell intrinsic cues but also impact tumor biology via modulation of the tumor microenvironment^29–31^. Along these lines, we found increased pPD-L1^Adj.^ levels in patients suffering from KRAS mutated NSCLC as compared to those with KRAS wildtype status (Fig. 7d). In contrast, mutations in EGFR, ALK fusions and ROS1 fusions or mutations showed no association with pPD-L1^Adj.^ levels, respectively (Fig. 7d, e).

We also explored a potential correlation of pPD-L1^Adj.^ with other clinical parameters. For example, we found that patients with higher tumor stages (T, *p* **=** 0.03), higher degrees of lymph node invasion (N, *p* **=** 0.04) and a higher tumor grading (G, *p* **=** 0.002) expressed more PD-L1 on the platelet surface (Fig. 7f–h). No association was found between pPD-L1^Adj.^ and the region of tumor origin (central vs. peripheral, Fig. 7i). However, pPD-L1^Adj.^ strongly correlated with the occurrence of metastases (*p* **<** 0.001), especially liver (*p* **=** 0.005) and bone metastasis (*p* **=** 0.001) (Fig. 7j–l). In line with previous studies^32^, we also found pPD-L1^Adj.^ to be positively correlated with smoking history and the amount of pack years (Supplementary Fig. 10c, d). Moreover, pPD-L1^Adj.^was positively correlated with platelet count, LDH and CRP (Supplementary Fig. 10j–l).

To further elaborate on the potential of pPD-L1^Adj.^ as a predictive biomarker in NSCLC, we conducted sequential measurements of pPD-L1^Adj.^ in 12 patients undergoing conventional chemotherapy or ICI (Supplementary Fig. 11a, b). Details on therapeutic regimens are provided in Supplementary Fig. 4. In these patients baseline pPD-L1^Adj.^ levels were determined prior to the first cycle of the respective 1st line treatment. The second measurement was conducted in parallel to the first CT scan. In patients treated with a platinum-based chemotherapy the tumor responses were quantified according to the Response Evaluation Criteria in Solid Tumors (RECIST) guidelines^33^. For patients receiving ICI iRECIST guidelines^34^ were used. In both groups response evaluation was performed 6–8 weeks after initial treatment. Remarkably, a significant drop in pPD-L1^Adj.^ levels was detected upon initiation of therapy in those patients whose tumors were later identified to have undergone at least partial remission (PR) (*p* **=** 0.02, Supplementary Fig. 11a). In contrast, patients who were later identified to have progressed despite therapy displayed a significant increase of pPD-L1^Adj.^ already in early measurements after therapy initiation (*p* **=** 0.04, Supplementary Fig. 11b). Of note, the predictive value of pPD-L1^Adj.^ was robust regardless of the used therapeutic regime. In two patients receiving ICI we determined pPD-L1^Adj.^ at multiple time points. Remarkably, pPD-L1^Adj.^ expression changes correlated with disease activity routinely determined via CT-scan (Supplementary Fig. 11c–f). As genomic alterations in EGFR and ALK represent independent factors influencing OS and progression-free survival (PFS), especially in patients receiving ICI, we additionally analyzed the role of pPD-L1^Adj.^ in the respective subgroups with or without such alterations. Whereas we were not able to detect a significant difference regarding OS (Supplementary Fig. 12a–c), in pPD-L1^Adj.^ high patients harboring an EGFR or ALK alteration who received a platinum-based chemotherapy, PFS tended to be worse compared to patients without EGFR and ALK alteration (Supplementary Fig. 12d, e). This might be explained by the fact that these patients had already shown a tumor progression upon first-line treatment with a tyrosine kinase inhibitor (TKI). In patients with EGFR and ALK alteration who received TKI, pPD-L1^Adj.^ was not predictive for PFS (Supplementary Fig. 12f). Since in our cohort none of the patients receiving ICI showed EGFR or ALK aberrations, the role of pPD-L1^Adj.^ could not be investigated in this cohort.

Finally, we set out to probe whether the pre-therapeutically determined pPD-L1^Adj.^ level can predict the therapy response of NSCLC patients to immune-checkpoint blocking antibodies. To do so we analyzed the PFS of patients either treated with only conventional chemotherapy or immunocheckpoint blockade. pPD-L1 positive and negative subgroups were defined according to the median pPD-L1^Adj.^ level. In patients receiving conventional chemotherapy we observed a significantly higher PFS when pPD-L1 levels were low (Fig. 7m). Interestingly, in patients treated with ICI (Pembrolizumab or Nivolumab), high pPD-L1^Adj.^ predicted a PFS benefit (HR 4.74, *p* **=** 0.003) (Fig. 7n and Supplementary Fig. 12j). Strikingly, when the predictive power of pPD-L1^Adj.^ was compared to conventional histological PD-L1 quantification (TPS **>** 50% and ≥1% in tumor biopsies), pPD-L1^Adj.^ was found to much better predict therapy response towards ICI (Fig. 7o, Supplementary Fig. 12k, l). In summary, our data suggest that pre-therapeutically measured pPD-L1^Adj.^ levels accurately predict the therapeutic response towards immune-checkpoint blocking antibodies. Prospective clinical trials are warranted to validate our findings and to justify the implementation of pPD-L1Adj as a biomarker in clinical routine.

## Discussion

Human cancers are heterogenous and biomarkers based on histopathological analyses of single tumor biopsies are often lacking robustness. Histological quantification of intratumoral PD-L1 expression is routinely performed on NSCLC biopsy material as an attempt to predict responses towards immune-checkpoint inhibition, however, the correlation between expression levels and the overall response rate (ORR) is limited^12^. In our present study we show that blood platelets are in frequent contact with lung cancer cells in vitro and in vivo and take up PD-L1 from the cancer cells in a fibronectin, integrin α5β1 and GPIbα dependent manner. Our data provides mechanistic explanation for recent reports describing PD-L1 on platelets from patients suffering from different types of cancers^32,35,36^. Interestingly, while there is comprehensive literature describing tumor cell induced platelet aggregation (TCIPA) and tumor cell-associated thrombus formation^37–41^, our herein presented data suggest that platelet-tumor cell contact can occur without substantial platelet activation and degranulation. Since pPD-L1 has not only been detected on the surface of activated platelets but also in resting platelets, it is tempting to speculate on an equilibrium between intracellularly stored pPD-L1 in α-granules and cell surface pPD-L1. Indeed, a similar mechanism has been described for the uptake and redistribution of fibrinogen and immunoglobulins^42^.

Importantly, as pPD-L1 is found to inhibit T cell function, it is likely that pPD-L1 plays a distinct role in systemic immunomodulation. Of note, pPD-L1 has recently been described in patients suffering from tumors which were classified as PD-L1 negative in biopsies^35^. Our herein presented data as well as other published studies on tumor heterogeneity suggest that immunohistochemistry-based quantification of protein expression on tissue sections from single biopsies should be interpreted with caution, as protein expression might differ spatially and temporally^43^. Obviously, while our herein presented data suggest a highly efficient uptake of PD-L1 from lung cancer cells into platelets, it does not exclude that some pPD-L1 might be derived from other sources such as endothelial or other non-malignant cell types.

As the total blood volume is circulated up to 1000 times through the body each day, we reasoned that platelets might mirror the collective PD-L1 payload of a tumor and thus might open up venues for novel biomarker strategies. In this regard it is striking that pPD-L1 not only correlates with tumor stage/grade and the occurrence of metastases but is found to be superior in predicting response towards immune-checkpoint inhibition when compared to standard histological PD-L1 quantification on tumor biopsies. Since in particular lung cancer represents one of the most frequent and lethal cancers worldwide^10^, further clinical investigation of pPD-L1 as a biomarker in NSCLC does not only hold the promise to unburden our health systems by avoiding costly and unnecessary therapies with ICI but, even more important, will avoid side effects of ICI in patients who would not benefit from this kind of therapy.

It should be mentioned that our exploratory study suffers from some limitations. Owing to the fact that we used an exploratory cohort of NSCLC patients with unequal representation of tumor stages and different cycles of various treatment regimens for the development of the calculation algorithm, pPD-L1^Adj.^ may not have yielded its maximum performance. Expectedly, while pPD-L1 can robustly detected in patients treated with TKI or platinum-based chemotherapy, pPD-L1 could not be detected in patients treated with anti-PD-L1 mAbs. Of note, this observation is in line with previous data^32^ and can be explained by binding of anti-PD-L1 mAbs to PD-L1 expressing platelets. However, this complicates an exact determination of pPD-L1 in these patients. Even if we did not observe a significant correlation of pPD-L1 and genetic alterations beyond KRAS, this exploratory study cohort of consecutively analyzed NSCLC patients might not be ideal to study the predictive role of pPD-L1 in NSCLC patients harboring genomic alterations including EGFR, ALK and ROS1.

Nevertheless, besides the tremendous potential of pPD-L1^Adj.^ as a biomarker, we believe that platelet PD-L1 might also represent a potential target for therapeutic intervention. This presumption is supported by our observation that pPD-L1 in NSCLC patients correlates with the number of T cells in TME and the number of infiltrating T cells. Similar observations in a mouse model support this finding^35^. Along these lines it is tempting to speculate that pPD-L1 might be involved in formation of the premetastatic niche by generating an immunotolerant environment at sites distant from the primary tumor (Supplementary Figs. 1, 2). Inhibition of pPD-L1 could prevent the formation of metastasis and such a concept would warrant the investigation of immune-checkpoint blocking antibodies in order to prevent metastasis when tumors with high metastatic risk are treated in a curative intention. Of note, clinical trials investigating the perioperative administration of ICI in NSCLC have reported reduced relapse and metastasis and our herein presented data might offer a mechanistic explanation for the observed results^44,45^. Last but not least, as pPD-L1^Adj.^ is shown to be prognostic and predictive in NSCLC, pPD-L1 might additionally serve as a liquid biomarker for early tumor detection or recurrence, an approach which warrants future clinical testing.

## Methods

### Study design and selection of patients

During 2016-2019, 173 consecutive patients with non-small lung cancer (NSCLC) treated in the Department of Medical Oncology and Hematology and Department of Internal Medicine VIII, University Hospital Tuebingen, Germany were prospectively included in the study (screening cohort = SC). In order to preclude the influence of anticoagulants like aspirin (ASS), low molecular weight heparin (LMWH) or other heparinoids and non‐vitamin K antagonist oral anticoagulants (NOACs), long‐term medication of each patient was considered. In our cohort 12 patients with LMWH and 28 patients taking ASS and/or clopidogrel were excluded. In Supplementary Fig. 4, a detailed flowchart of patient selection is given. In all cases, sample collection was performed prior to the next application of the respective therapy. Tumor characteristics are based on baseline clinical staging. In order to take disease progression better into account the occurrence of metastasis was double checked at the time point of study inclusion. Our cohort comprised 71 male and 57 female patients with a mean age of 65.7 years (range 19–87). The diagnosis of a NSCLC was histologically confirmed in all cases. NSCLC adenocarcinoma was identified in 93 patients (72.7%), in 35 cases (27.3%) a squamous cell carcinoma was found. The details of the all patients’ characteristics are summarized in Supplementary Table 1. Written informed consent was given in all cases. Sample collection of healthy participants were in accordance with the ethical standards of the institutional research committee (Ethic committee of the Faculty of Medicine of the Eberhard Karls University Tuebingen and of the University Hospital Tuebingen vote 13/2007V). The observational study in NSCLC patients was approved by the ethics committee of the Faculty of Medicine of the Eberhard Karls University Tuebingen and of the University Hospital Tuebingen and was conducted in accordance with the Declaration of Helsinki (vote 456/BO2).

### Preparation of platelets

Platelets were obtained from healthy donors (not taking any medication for at least 10 days) and NSCLC patients after informed writing consent. Citrated blood was briefly centrifuged for 20 min at 120 × *g*, the upper fraction was harvested as platelet-rich plasma (PRP). Platelets were washed twice with citrate wash buffer (128 mmol/L NaCl, 11 mmol/L glucose, 7.5 mmol/L Na_2_HPO_4_, 4.8 mmol/L sodium citrate, 4.3 mmol/L NaH_2_PO_4_, 2.4 citric acid, 0.35% bovine serum albumin, and 50 ng/mL prostaglandin E_1_ (PGE1)). To avoid the influence of PGE1 on platelet-tumor cell and platelet-immune cell interaction, we did not use PGE1 in our co-incubation experiments. For platelet activation 10 µM of the Thrombin Receptor Activator Peptide 6 (TRAP-6), a protease-activated receptor 1 (PAR_1_) agonist, 2.5 µM ADP or 5 µg/mL Collagen was added to the platelets for 2 min. Platelets were fixed by 2% paraformaldehyde for 10 min and washed twice with PBS containing 1% FCS.

### Flow cytometry

Flow cytometry was performed using fluorescence-conjugates or specific mAb and their controls followed by species-specific conjugate (Supplementary Table 2) using a FACS CantoII flow cytometer (Beckman Coulter) or a LSRFortessa (Becton Dickinson) from the flow cytometer facility Tuebingen. Positive cells in percentage (%) were calculated as follows: Surface expression in percent obtained with the specific antibody—surface expression in percent obtained with isotype control. Platelets were preselected by CD41a^+^ and CD62P^−^ (resting) or CD62P^+^ (activated). Data analysis was performed using FlowJo software (v.10). In order to verify the reproducibility of our flow cytometry system, we performed a Bland–Altman analysis (Supplementary Fig. 8f). For immunophenotyping of PBMC subsets of lung cancer patients and healthy control donors were identified by counterstaining with CD3-PECy5 (BD biosciences, San Diego, CA), CD19-APC/Fire750, CD4-Pacific Blue, CD8a-BV605, CD56-PECy7, CD14-BV785, HLA-DR-BV650 (Biolegend, San Diego, CA) and CD16-FITC (invitrogen). PD-1 and PDL-1 expression as well as activation levels were analyzed using a PD-1-APC or PDL-1-APC and a CD69-PE antibody (BD biosciences), respectively. Isotype controls were obtained from BD biosciences. Dead cells were excluded from analysis with LIVE/DEAD™ Fixable Aqua (Thermo Fisher Scientific, Waltham, MA).

### Histopathology, immunohistochemistry and immunofluorescence staining of paraffin-embedded tissue samples

Tissue samples were fixed in 4% formalin and paraffin-embedded (FFPE) at the Department of Pathology (University Hospital Tuebingen). The sections were cut briefly in 3 µm sections and stained with Hematoxylin/Eosin and CD61 (clone: 2C9.G3) following standard protocols. For immunofluorescence microscopy, sections were deparaffinized and hydrated in a first step. The heat-induced antigen retrieval method was performed using sodium citrate buffer (pH 6.0) for 30 min. Antigen blocking was performed with Blocking solution (Zytomed) for 60 min. Primary antibodies that were included anti-CD41, mouse, 1:250 (clone: HIP8) and anti-PD-L1, rabbit, 1:200 (clone:28-8). Secondary antibodies include Alexa-Fluor 594 labeled anti-rabbit (1:1000, Invitrogen) and Fluor 488 labeled anti-mouse (1:1000, Invitrogen). DAPI (1:1000, BioLegend) was used for nuclear staining prior mounting the slides with H-1500 Vectashield Hardset. Microscopic analysis was done with an Olympus BX63 microscope and a DP80 camera (Olympus).

### Immunofluorescence staining of platelets and tumor cells

For immunostaining tumor cells and/or platelets were fixed in 2% PFA in PBS (pH 7.4) for 10 min at −20 °C. After three washing steps in PBS cells were incubated with a BSA blocking solution (2% BSA, 0,2% Triton X-100, 0,1% Tween) for 1 hour. Primary antibodies were anti-PD-L1, rabbit (1:250, clone: 28-8), anti-CD41, mouse (1:400, clone: HIP8), anti-CD61, rabbit (1:250, clone: SJ-19-09), anti-GFP, rabbit (1:200, clone: EPR14104), anti-fibronectin, mouse (1:200, clone: P1H11); as secondary antibodies Alexa-Fluor 488/594 labeled anti-rabbit (1:1000, Invitrogen) and Fluor 488/594 labeled anti-mouse (1:1000, Invitrogen) were used. Afterwards slides were mounted in fluorescent mounting medium containing DAPI (1:1000, BioLegend) counter-stain. For the plasma membrane staining CellMask^TM^ (ThermoFisher) and Dil (ThermoFisher) were used according to manufactures' instructions. For nuclear staining NucBlue^TM^ (ThermoFisher) was used. Image acquisition was performed using an Olympus BX63 microscope and a DP80 camera (Olympus) and CellSens Dimension 1.17 software. Quantification of platelets, fibronectin, and tumor cells were performed via counting fluorescence positive signals using an ImageJ script (v.1.51n and v.1.52).

### Cyclic immunofluorescence staining of NSCLC patient samples

Paraffin-embedded patient samples were cut in 2–5 µm slices and collected on object slides. Subsequently, sections were subjected to deparaffinization and rehydration. Slides were treated with xylene for 10 min, followed by rehydration using an ethanol dilution series of 100%, 95%, 70%, 50% for 5 min each. One last change was performed using deionized water. Heat-induced antigen retrieval was performed using a Sodium-Citrate buffer (10 mM Sodium citrate, 0.05% Tween 20, pH 6.0) and boiling the samples for 20 min. Samples were cooled down and stored in MACSima™ Running Buffer (Miltenyi Biotec, 130-121-565) until initial DAPI staining (Miltenyi Biotec, 130-111-570). The MACSima^TM^ device is an ultra-high content cyclic IF device which allows for fully automated IF imaging. Iteratively, the device performs fluorescent staining with multiple labeled antibodies, image acquisition, and bleaching per cycle. Images were generated according to the manufacturer’s instructions and analyzed with the Qi Tissue Image Analysis Software. For quantification at least two ROI were selected based on manual prestaining of DAPI.

### Tracking platelet-tumor cell interaction using live-cell imaging

For live-cell imaging analysis A549 cells (cultured as stated above) were used. Tumor cells were co-incubated with platelets at a platelet-tumor cell ratio of 1:1000. Platelets were added to the tumor cells directly prior image acquisition. Platelet-tumor cell interactions were analyzed using phase-contrast live-cell microscopy with frame intervals of 30 s for up to 40 min (Leica Microsystems, Thunder Imager 3D Assay; HC PL APO 40 × /0.95) using adaptive focus control. Cell positions were assigned by their center-of-mass coordinates.

### Electron microscopy and immunoelectron microscopy

For transmission electron microscopy, platelets from one representative pPD-L1 high expressing NSCLC patient were used. Platelets were centrifuged and the resulting pellets were fixed for 24 h in Karnovsky’s fixative. As previously described, Ultrathin sections were examined with a LIBRA 120 (Zeiss) operating at 120 kV^46^. For immunoelectron microscopy, platelets were fixed and embedded in Lowicryl K4M (Polysciences)^47^. Samples were stained with anti-PD-L1 antibody (Abcam) and examined using a LIBRA 120 transmission electron microscope (Zeiss) at 120 kV.

### ELISA

Protein levels of PD-L1 were measured using a human PD-L1 ELISA kit (Abcam, clone: 28-8) according to the recommendations of the manufacturer. All concentrations are expressed as means ± SEM of triplicates.

### Western blot

Whole-cell extracts were prepared using RIPA buffer and protein concentration was analyzed using the BioRad Dc assay. 25-50 µg of protein were transferred to 10-15% SDS-Page and blotted on a PVDF membrane (Millipore) with a wet blot system. The membrane was blocked for 1 h at room temperature with Roti-Block, followed by overnight incubation with the following antibodies: anti-PD-L1, rabbit (1:2000, clone: 28-8), anti-fibronectin, mouse (1:250, clone: P1H11), anti-Vinculin, mouse (1:10,000, clone: hVIN-1), anti-α tubulin (1:10,000, clone 11H10) and anti-ß Actin (1:10,000, clone AC-15). Blots were visualized using ECL reagents (GE Healthcare) or the Super Signal West Kit (Thermo Scientific) and the ChemiDocTM MP Imaging System using the ImageLab v5.2.1 software.

### Real-time PCR

To determine mRNA abundance in several tumor cell lines we extracted mRNA in TriFast (Peqlab) according to the manufacturer’s instructions. After DNAse digestion reverse transcription of total RNA was performed using random hexamers (Roche Diagnostics) and SuperScriptII reverse transcriptase (Invitrogen). Amplification of the respective genes by real-time polymerase chain reaction (RT-PCR) was performed in a total volume of 20 μl using 40 ng of cDNA, 500 nM forward and reverse primer and 2x GoTaq® qPCR Master Mix (Promega) according to the manufacturer’s protocol. Cycling conditions were as follows: initial denaturation at 95 °C for 2 min, followed by 40 cycles of 95 °C for 15 s, 55 °C for 15 s and 68 °C for 20 s. For amplification the following primers were used (5′->3′orientation): Fibronectin (FN1), fw ACCGTGGGCAACTCTGTCAA, rev CCCACTCATCTCCAACGGCA; Tissue factor (F3), fw GGCACGGGTCTTCTCCTACC, rev TGTCCGAGGTTTGTCTCCAGG; Von Willebrand Factor (VWF), fw CCTGCACCGACATGGAGGAT, rev CGTAAGTGAAGCCCGACCGA; Fibrinogen A (FBG), fw TGAAACGACTGGAGGTGGACA, rev CACGAGCTAAAGCCCTACTGC; GAPDH (GAPDH), fw TCGACAGTCAGCCGCATCTT, rev GCCCAATACGACCAAATCCGT. Real-time PCR amplifications were performed on a CFX96 Real-Time System (Biorad). All experiments were performed in duplicates and analyzed via the 7500 Software v2.0.6. The housekeeping gene GAPDH was used to standardize the amount of sample RNA.

### In vitro platelet-tumor cell co-incubation and platelet adhesion

Tumor cells were coated with platelets as described previously with slight modifications^41,48^. Briefly summarized, PRP was obtained from fresh whole blood by centrifugation for 20 min at 120 g. Platelets were washed twice with citrate wash buffer (128 mmol/L NaCl, 11 mmol/L glucose, 7.5 mmol/L Na_2_HPO_4_, 4.8 mmol/L sodium citrate, 4.3 mmol/L NaH_2_PO_4_, 2.4 citric acid, 0.35% bovine serum albumin). In some experiments platelets were pre-treated with 5 µg/mL anti-CD42b (clone: AK2), 20 µg/mL anti-integrin ß1 (clone: P4C10) and anti-Integrin α5 (clone: JBS5) or corresponding control IgG_1_ (20 µg/mL) for 30 min at 37 °C and 7% CO_2_. Tumor cells were incubated in platelets at a platelet-tumor cell ratio of 1:1000 for 30 min at 37 °C and 7% CO_2_. For immunofluorescence microscopy and FACS analysis cells were fixed in 2% PFA in PBS (pH 7.4) for 10 min at −20 °C prior staining.

### Preparation of fibronectin matrices and platelet blocking

To prepare fibronectin matrices, plates were coated with a human plasma fibronectin purified protein (R&D, Minneapolis, MN, USA) (concentration 50 µg/cm²) for 120 min. For blocking of GPIb-IX-V complex, α5ß1 or GPIIbIIIa, washed platelets (8 × 10^7^/mL) were pre-treated with 5 µg/mL anti-CD42b (clone: AK2), 20 µg/mL anti-integrin ß1 (clone: P4C10) and anti-Integrin α5 (clone: JBS5), 1 µg/mL Tirofiban or corresponding control IgG_1_ (20 µg/mL) for 30 min at 37 °C and 7% CO_2_. After co-incubation with platelets (8 × 10^7^/mL) for 30 min at 37 °C and 7% CO_2_, non-adherent platelets were removed via three washing steps using PBS. After removal of non-adherent platelets cells were fixed in 2% PFA in PBS (pH 7.4) for 10 min at −20 °C. Platelet adhesion to fibronectin fibrils was evaluated by calculating surface coverage area and platelet count/FoV and from microscopic images using an ImageJ script (v.1.52).

### Plasmid construction, transfection and knockdown of NSCLC cells

For overexpression of PD-L1 (CD274) a True-ORF-GFP-tagged expression vector was used (OriGene, RG213071, Rockville, MD, USA). Control cells were transfected using a FLAG tag. The FLAG cDNA was generated by PCR and cloned into the PD-L1-GFP vector using AsiSI and MluI restriction sites. Data analysis was done using ApEv.2.0.51. Tumor cells were transfected with 2.5 µg DNA (PD-L1-GFP, FLAG-GFP) using Lipofectamine^TM^ 3000, in accordance with the manufacturer’s instructions. For siRNA knockdown of Fibronectin, Lipofectamine^TM^ 3000 and 100 pmol of human (FN1) siRNA Oligo Duplex (Locus ID 2335) (Origene, SR320193) was used. As scrambled negative control we used 100pmol universal scrambled negative control siRNA (Origene, SR30004).

### Generation of peripheral blood mononuclear cells (PBMC) and tumor cell lines

Peripheral blood mononuclear cells (PBMC) from healthy donors were isolated using Ficol/Paque (Biochrom) density gradient centrifugation after informed consent. All tumor cell lines were cultured with 10% FCS in Roswell Park Memorial Institute (RPMI) 1640 Medium at 37 °C and 7% CO_2_. Cell proliferation was quantified using a Neubauer chamber; for viability testing Trypan blue staining’s was performed using a 0.4% trypan blue solution (Fluka). The tumor cell lines A549 (CRM-CCL-18), NCI-H460 (HTB-177), NCI-H23 (CRL-5800), NCI-H226 (CRL-5826), NCI-H322 (CRL-5806), NCI-H522 (CRL-5810), HOP-62 and HOP-92 were obtained from the American Type Culture Collection (ATCC). Mycoplasma contamination was excluded via a PCR-based method.

### IFNγ ELISPOT assay in CD8^+^ T cells and platelet T-cell co-incubation

Freshly thawed (ex vivo) PBMCs from healthy donors were analyzed by enzyme-linked immunospot (ELISPOT) assay in duplicates. Interferon γ (IFNγ) ELISPOT assays in our study were performed as described previously^49^. In brief, 96-well nitrocellulose plates (Millipore) were coated with 1 mg/mL anti-IFNγ mAb (Mabtech) and incubated overnight at 4 °C. In a next step, plates were blocked with human serum (10%) for 2 hours at 37 °C. PBMCs (2.5 × 10^5^ cells per well) were pulsed with an EBV/CMV epitope mix containing the frequently recognized peptides BRLF109-117 YVLDHLIVV (A*02) peptide and CMV pp65 (A*02) peptide NLVPMVATV and incubated with or without platelets (ratio 1:50) for 24 h. Phytohemagglutinin was used as positive control. HLA-A*02 (KLFEKVKEV)- and B*07 (KPSEKIQVL)-restricted control peptides derived from benign tissues (HV-exclusive HLA ligands) served as negative control. Prior co-incubation with T cells PD-L1 positive platelets from NSCLC patients were pre-treated with the anti-PD-L1 mAB Atezolizumab for 30 min and washed twice with PBS containing 1% FCS. Readout was performed according to the manufacturer’s instructions. Spots were counted using an ImmunoSpot S5 analyzer (CTL).

### Cytokine and cell surface marker staining

Peptide-specific T cells were further analyzed by intracellular cytokine and cell surface marker staining. PBMCs were incubated with 10 μg ml^−1^ of peptide, 10 μg ml^−1^ brefeldin A (Sigma-Aldrich) and a 1:500 dilution of GolgiStop (BD) for 12–16 h. Staining included Cytofix/Cytoperm solution (BD), anti-CD4, mouse (1:100, clone: RPA-T4), anti-CD8, mouse (1:400, clone: B9.11), anti-TNF, mouse (1:120, clone: Mab11) and anti-IFN-γ, mouse (1:200 dilution, clone: 4SB3). PMA (5 μg ml^−1^) and ionomycin (1 μM, Sigma-Aldrich) served as positive control. Viable cells were determined using Aqua live/dead (1:400 dilution, Invitrogen). Samples were analyzed on a FACS Canto II cytometer (BD) and evaluated using FlowJo software v.10.0.8 (BD).

### Generation of NY-ESO-1-specific CD4^+^ T cells and platelet T-cell co-incubation

The generation of NY-ESO-1-specific T cells was performed using as described previously^50^. In brief, PBMCs from a healthy donor (1 × 10^7^/mL) were stimulated using pools of NY-ESO-1 overlapping peptides (1 µg/mL). The NY-ESO-1 overlapping peptide pool of 15 amino acid length (11 amino acid overlap) was purchased via Miltenyi Biotec. The cells were cultured in RPMI 1640 containing 10 % human AB-serum and 1% l-glutamin in the presence of 10 U/mL recombinant IL-2 and 10 ng/mL Il-7. Culture medium was replaced every third day. After a pre-sensitization period of 7-14 days, NY-ESO-1 specific, IFNγ^+^ T cells were enriched after re-stimulation with NY-ESO-1 peptide pool for 6 h using CliniMACS® (Miltenyi Biotec) technique as reported previously^51^. After enrichment, NY-ESO-1 specific T cells were expanded for 14 days in the presence of IL-7 (10 ng/mL), IL-15 (10 ng/mL) and IL-2 (50 U/mL). T cell specificity was analyzed via intracellular IFNγ staining as stated above. For further characterization of the T-cells the differentiation markers anti-CD45RO, mouse (1: 200, clone: HI100), anti-CD62L, mouse (1:400, clone: DREG-56), anti-CD28, mouse (1:200, clone: CD28.2) and anti-CD27, mouse (1:200, clone: M-T271) were co-analyzed by flow cytometry. For the platelet-T-cell co-incubation assay, NY-ESO-1 specific T cells (5 × 10^6^/mL) were cultured in TexMACS GMP Medium (Miltenyi Biotec). Six hours prior analysis T cells were co-incubated with platelets of NSCLC patients or healthy donors (ratio 1:200) and re-stimulated with NY-ESO-1 peptides (1 µg/mL). In order to investigate the functional role of PD-L1 on platelets surfaces, PD-L1 positive platelets from NSCLC patients were pre-treated with Atezolizumab (100 µg/mL) for 30 min and washed twice with PBS containing 1% FCS. As a negative control a Myelin oligodendrocyte glycoprotein (MOG) peptide mix was used (1 μg/mL). SEB (Toxin Technology, Sarasota, FL, USA) at 10 µg/mL was used as positive control. NY-ESO-1 specific T cell activity was determined by intracellular TNFα and IFNγ quantified via flow cytometry as described above.

### In situ proximity ligation assay (PLA)

HOP-62 and NCI-H23 cells were grown on glass bottomed plates. After two washing steps cells were fixed in 1% PFA in PBS (pH 7.4) for 10 min at −20 °C. After three washing steps in PBS cells were incubated with a BSA blocking solution (5% BSA, 0,2% Triton X-100, 0,1% Tween) for 30 min. In situ PLA was performed using the Duolink PLA kit (Sigma-Aldrich) according to the manufacturer’s instructions. In brief, after blocking cells were incubated with anti-PD-L1, rabbit (1:250, clone: 28-8) and anti-fibronectin, mouse (1:200, clone: P1H11) for 2 h at room temperature. After three washing steps with PBST (phosphate buffered saline, 0.1% Tween), anti-mouse PLUS and anti-rabbit MINUS PLA probes were linked to the primary antibodies for 1 h at 37 °C. After three times washing steps with buffer A (0.01 M Tris, 0.15 M NaCl, and 0.05% Tween-20), PLA probes were ligated for 60 min at 37 °C. After two washing steps with buffer A, amplification using Duolink In Situ Detection Reagents (Sigma) was performed at 37 °C for 120 min. Following amplification, cells were washed three times for 5 min with wash buffer B (0.2 M Tris 0.1 M NaCl). Cells were then coated with Duolink Mounting Medium containing DAPI. Image acquisition was performed using an Olympus BX63 microscope and a DP80 camera (Olympus).

### Establishment of an activation-independent calculation matrix for platelet PD-L1

Since platelet pre-activation levels differ due to sample collection/preparation and protein surface expression depends on the platelet activation state, accurate determination of total protein expression on platelet surfaces is challenging. As a result, the platelet pre-/activation level acts as a confounding factor and thus impairs the suitability of pPD-L1 as a promising biomarker in NSCLC. To circumvent this dilemma, we established an activation-independent calculation matrix of platelet PD-L1. The matrix is based on our cohort of 128 NSCLC patients and investigates the activation-dependent expression change of PD-L1 (ΔpPD-L1) during controlled platelets stimulation ex vivo. Patients were categorized into pPD-L1 quartile groups (Q1: very low, Q2: low, Q3: medium, Q4: high), according to the pPD-L1 expression in unstimulated platelets. The pre-activation of platelets after sample preparation was determined via CD26P expression. In a second step each quartile group was subdivided according to the respective pre-activation levels (CD62P expression: 0–20%, 20–40%, 40–60%, 60–80%, and 80–100%) according to the pre-activation levels. The activation-dependent expression changes of PD-L1 (ΔpPD-L1) was then calculated for each subgroup. An overview of the subsampling and calculation is given in Supplementary Fig. 4.

### Statistics

Student’s *t* test, Mann–Whitney *U* test, one‐way ANOVA and Friedman’s test were used for continuous variables, chi‐squared test or Fisher’s exact test for categorical variables. If significant differences by one-way ANOVA were found, group wise comparison was done (Tukey’s multiple comparison test). If significant differences by Friedman’s test were found Dunn’s multiple comparisons test was used. Prior performing each statistical test we tested for normal distribution using the D’Agostino & Pearson test. Overall survival (OS) and progression-free survival (PFS), including the median, were calculated using the Kaplan‐Meier method. Hazard ratios (HRs) were determined using Cox regression analysis. OS was calculated from the date of primary diagnosis or time-point of study inclusion and stratified by the end of the study. The predictive value of platelet-derived PD-L1 as a prognostic factor was evaluated by examining the area under the receiver‐operator characteristic (ROC) curve using a confidence interval of 95%. All statistical tests were considered statistically significant when *P* was below 0.05. Statistical analysis was performed using SigmaStat, SPSSv21 and v27 and GraphPadPrism (v.8.1.0 and v.8.4.0).

### Reporting summary

Further information on research design is available in the Nature Research Reporting Summary linked to this article.

## Supplementary information

Supplementary Information

Reporting Summary

Description of Additional Supplementary Files

Supplementary Movie 1

Supplementary Movie 2

## Data Availability

All data generated in this study are available within the Article, Supplementary Information or Source Data file. Source data are provided with this paper.

## Source data

Source Data
