# Supplementary material for: Platelet PD-L1 reflects collective intratumoral PD-L1 expression and predicts immunotherapy response in non-small cell lung cancer
Source: Nat Commun. 2021 Dec 1;12:7005. doi: 10.1038/s41467-021-27303-7 (PMC8636618; doi:10.1038/s41467-021-27303-7)
Supplement: Supplementary file 1 — Supplementary Information [file 41467_2021_27303_MOESM1_ESM.pdf]

Supplementary Information:

Platelet PD-L1 reflects collective intratumoral PD-L1 expression and predicts immunotherapy response in non-small cell lung cancer.

Clemens Hinterleitner[1,2,] Jasmin Strähle[3], Elke Malenke[4], Martina Hinterleitner[1,2], Melanie Henning[1,2], Marco Seehawer[1,2], Tatjana Bilich[2,5,6], Jonas Heitmann[2,6], Martina Lutz[2,6], Sven Mattern[7], Sophia Scheuermann[2,4], Marius Horger[8], Stefanie Maurer[2,6,9], Juliane Walz[2,5,6], Falko Fend[7], Rupert Handgretinger[2,4], Christian Seitz[2,4], Bettina Weigelin[10], Stephan Singer[7], Helmut Salih[2,6], Oliver Borst[11], Hans-Georg Kopp[12], Lars Zender[1,2,13, #]

[1] Department of Medical Oncology & Pneumology (Internal Medicine VIII), University Hospital Tuebingen, Tuebingen, Germany

[2] DFG Cluster of Excellence 2180 'Image-guided and Functional Instructed Tumor Therapy' (iFIT), University of Tuebingen, Tuebingen, Germany

[3] Department of Haematology, Oncology and Immunology, University Hospital Tuebingen, Tuebingen, Germany

[4] Department of Pediatric Hematology and Oncology, University Hospital Tuebingen, Tuebingen, Germany

[5] Institute for Cell Biology, Department of Immunology, University of Tuebingen, Tuebingen, Germany

[6] Clinical Collaboration Unit Translational Immunology, German Cancer Consortium (DKTK), Department of Internal Medicine, University Hospital Tuebingen, Tuebingen, Germany

[7] Department of Pathology and Neuropathology, University Hospital Tuebingen, Tuebingen, Germany

[8] Department of Radiology, University Hospital Tuebingen, Tuebingen, Germany

[9] Department of Radiology, Memorial Sloan Kettering Cancer Center, New York, NY, USA

[10] Werner Siemens Imaging Center, Department of Preclinical Imaging and Radiopharmacy, Eberhard Karls University Tuebingen, Tuebingen, Germany

[11] University Hospital, Department of Cardiology and Angiology, Eberhard Karls University of Tuebingen, Tuebingen, Germany

[12] Robert-Bosch-Hospital, Department of Molecular and Pneumological Oncology, Stuttgart, Germany

[13] German Cancer Research Consortium (DKTK), Partner Site Tübingen, German Cancer Research Center (DKFZ), 69120 Heidelberg, Germany

[#] To whom correspondence should be addressed

List of Items

Supplementary Figure 1: Proposed model of pPD-L1 transfer from tumor cells to platelets and immunologic functions of pPD-L1.

Supplementary Figure 2: Proposed model of the role pPD-L1 in the microenvironment and potential therapeutic interventions.

Supplementary Figure 3: Direct platelet-tumor cell interactions but not supernatant enables protein exchange.

Supplementary Figure 4: Flow chart of patient selection.

Supplementary Figure 5: PD-L1 expression on platelets.

Supplementary Figure 6: Immunomodulation of platelets derived from healthy donors.

Supplementary Figure 7: Immunophenotyping in NSCLC patients.

Supplementary Figure 8: Association of pPD-L1 expression and platelet activation in healthy donors.

Supplementary Figure 9: Algorithm of pPD-L1Adj calculation.

Supplementary Figure 10: Correlation of pPD-L1Adj and clinical parameters.

Supplementary Figure 11: pPD-L1 predicts treatment response in NSCLC.

Supplementary Figure 12: pPD-L1 as prognostic and predictive marker in NSCLC.

Supplementary Figure 13: Representative FACS gating strategies.

Supplementary Table 1: Patients characteristics of the proof of principle (PoP) cohort.

Supplementary Table 2: Antibodies

Supplementary Figures

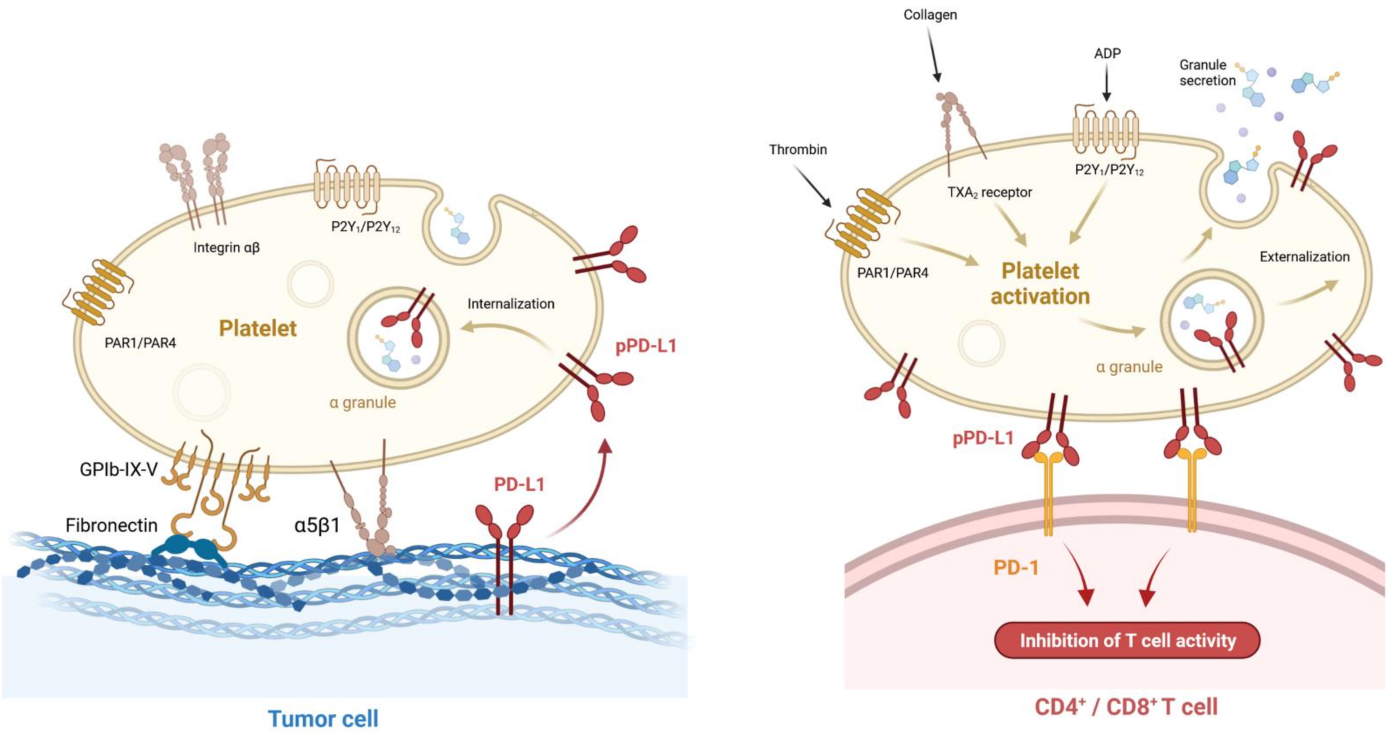

Supplementary Figure 1: Proposed model of pPD-L1 transfer from tumor cells to platelets and immunologic functions of pPD-L1. Tumor cells expressing fibronectin enhance tumor cell platelet crosstalk mediated via platelet GPIb-IX-V and integrin α5β1 and lead to a consecutive uptake of PD-L1 from the tumor cell. In platelets, pPD-L1 is expressed on the platelet surface and stored intracellularly in α-granules. Platelet activation via thrombin, ADP or collagen increase pPD-L1 expression on the platelet surface and finally suppress T cell reactivity. The graphic was created using BioRender (BioRender.com, Toronto, Canada).

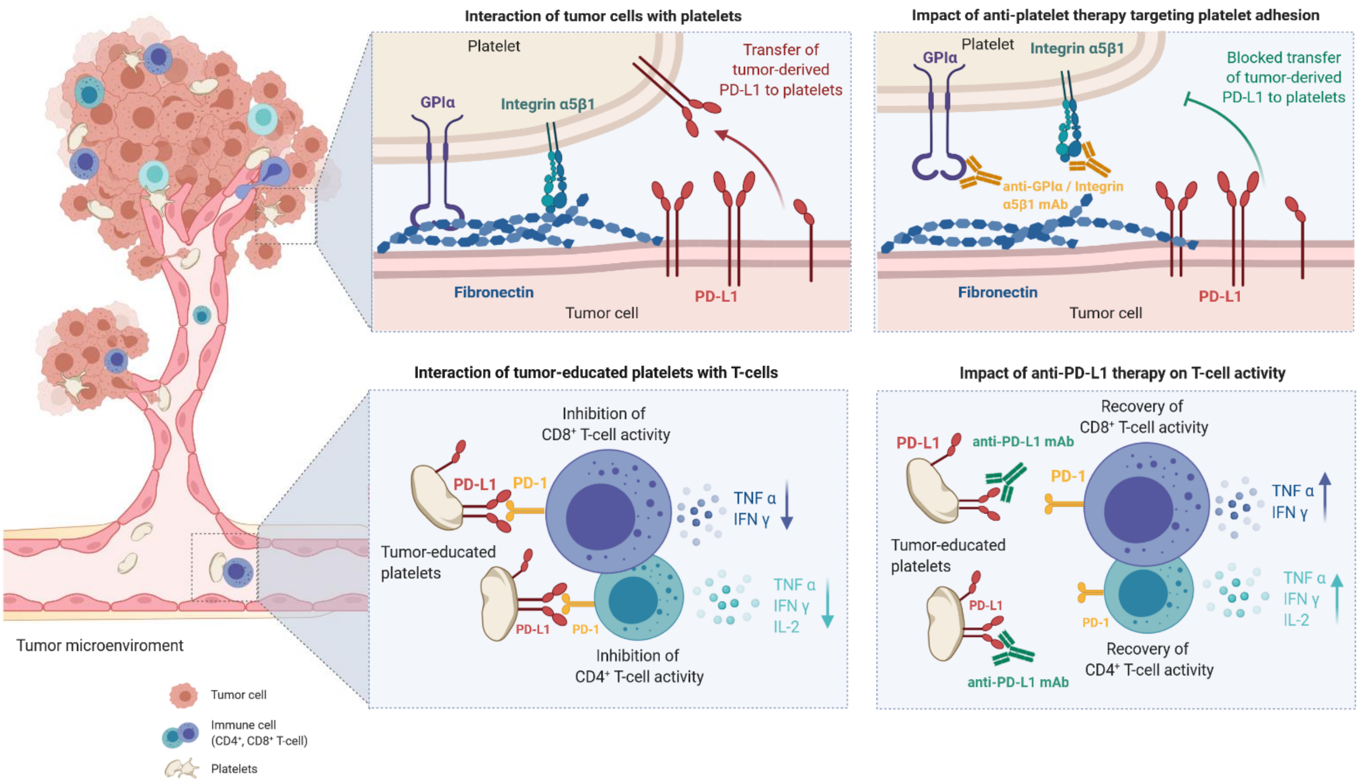

Supplementary Figure 2: Proposed model of the role pPD-L1 in the microenvironment and potential therapeutic interventions. In the TME platelets frequently interact with tumor cells and ingest tumor PD-L1 in a fibronectin, GPIb-IX-V and integrin α5β1 dependent manner. Blocking of GPIb-IX-V and integrin α5β1 reduce the uptake of PD-L1 and lower the immune inhibitory capacity of platelets in the TME. Blocking of pPD-L1 via anti-PD-L1 mAbs might contribute to the recovery of the anti-tumor T cell activity. The graphic was created with BioRender software (BioRender.com, Toronto, Canada).

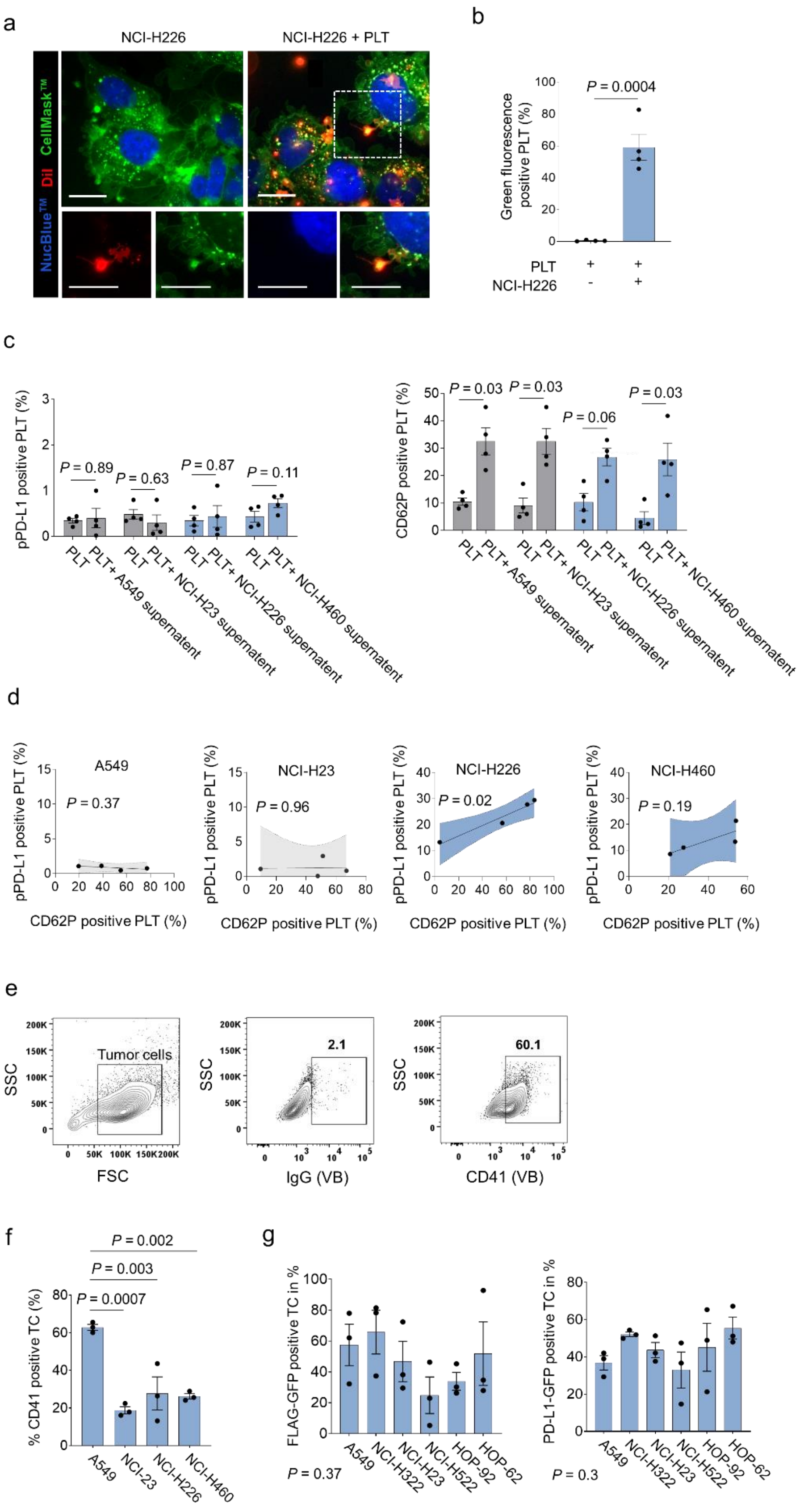

Supplementary Figure 3: Direct platelet-tumor cell interactions but not supernatant enables protein exchange. a Membrane transfer of NCI-H226 cells with platelets. Immunofluorescence of lipid membranes (CellMaskTM) in NCI-H226 cells in green. Platelets were counter-stained using Dil (red). Nuclei were stained using NucBlueTM (n = 4). Scale bars, 20 µm. b Percentage of green fluorescence positive platelets after co-incubation with green labelled NCI-H226 cells quantified via flow cytometry (n = 4). Data are mean ± SEM. Statistical significance was calculated by two-tailed Student's t-test.

c Surface expression of PD-L1 and CD62P on control platelets (PLT) and platelets after co-incubation with supernatant derived from A549, NCI-H23, NCI-H226, NCI-H460 cells (n = 4). Data are mean ± SEM. Statistical significance was calculated by two-tailed Student's t-test. d Correlation of pPD-L1 expression and platelet activation (CD62P expression) on platelets after co-incubation with A549, NCI-H23, NCI-H226, NCI-H460 cells (n = 4). Correlation was determined by simple linear regression analysis. e Flow cytometry gating strategy for the quantification of platelets bound to A549 tumor cells after co-incubation. f Quantification of platelets bound to tumor cells (n = 3). Data are mean ± SEM. Statistical significance was calculated by two-tailed Student's t-test. g Quantification of the transfection efficacy of FLAG-GFP and PD-L1-GFP transfected tumor cells (n = 3). Data are mean ± SEM. Statistical significance was calculated by one-way ANOVA and Tukey's multiple comparisons test. Source data are provided as a Source Data file.

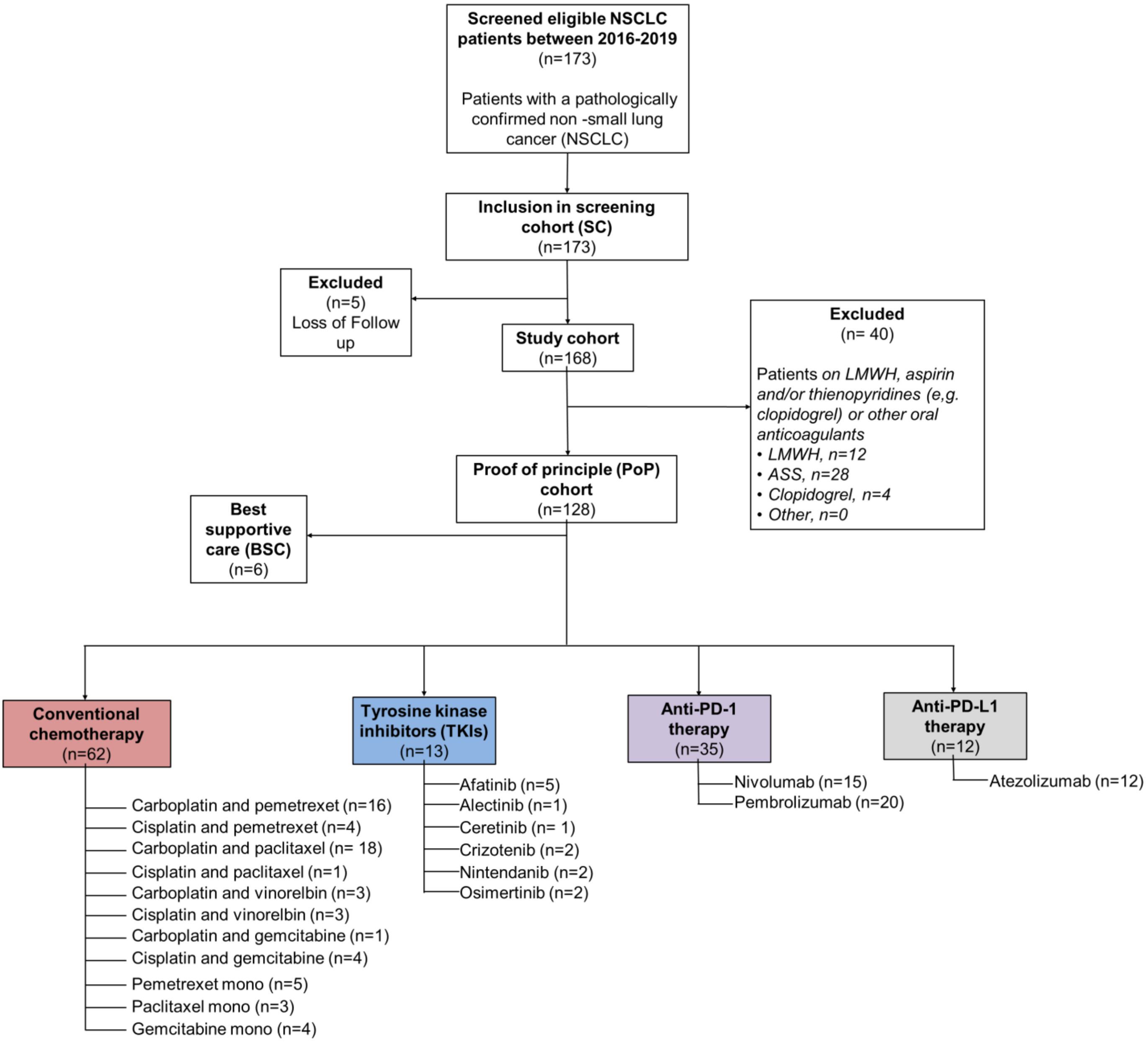

Supplementary Figure 4: Flow chart of patient selection. During 2016-2019, 173 patients were screened for eligibility. 173 patients were included in the screening cohort (SC). 40 patients talking anticoagulating agents were excluded from further analysis. 128 patients were finally included in the proof of principle cohort (PoP).

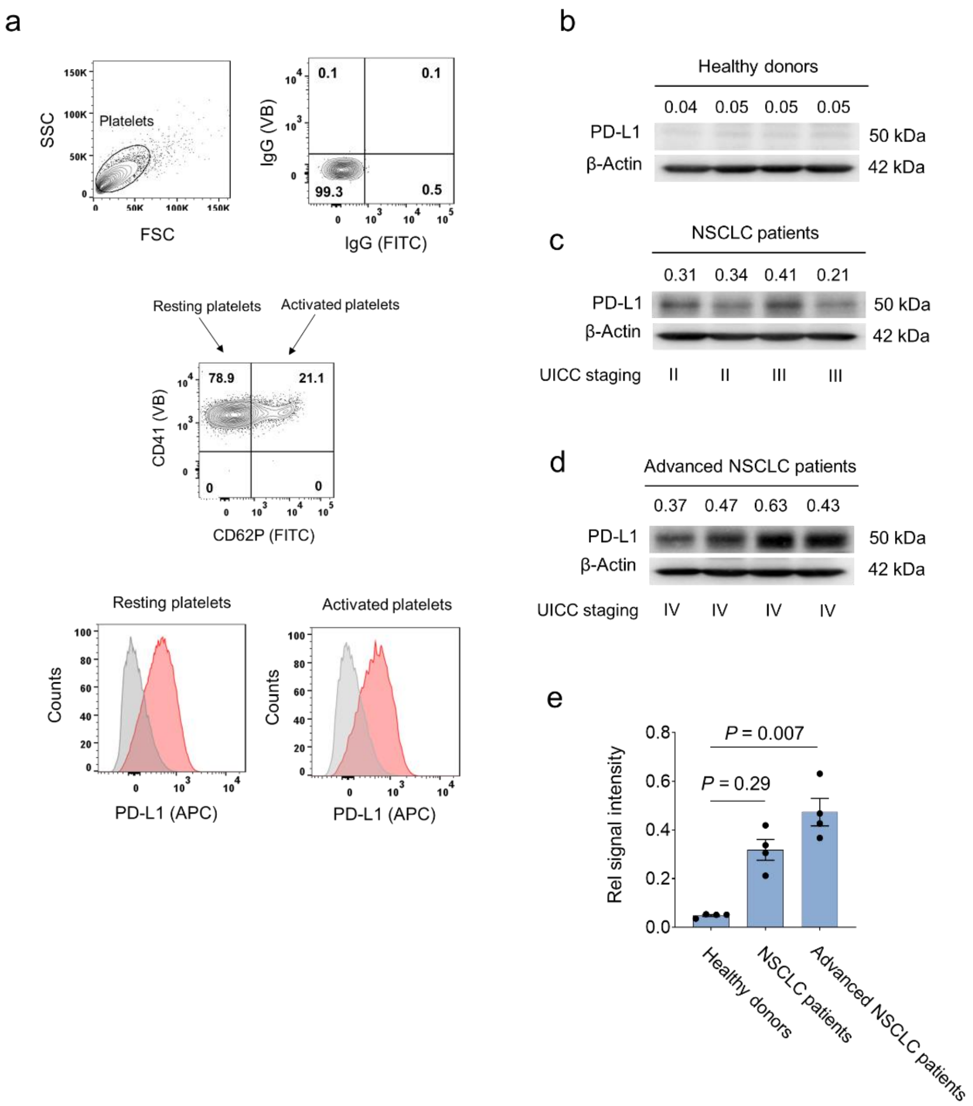

Supplementary Figure 5: PD-L1 expression on platelets. a Flow cytometry gating strategy showing the PD-L1 expression on resting and activated platelets of a NSCLC patient (n = 3). b Western blots analysis of platelet whole cell lysates showing PD-L1 in healthy donors. ß-actin was used as loading control (n = 4). c-d Western blots analysis showing PD-L1 in NSCLC patients with intermediate (c) and high tumor stages (d) (n = 8). e Quantification of PD-L1 in platelet whole cell lysates of heathy donors (n = 4) and NSCLC patients (n = 8) out of one independent experiment. Data are mean ± SEM. Statistical significance was calculated by Kurskal-Wallis test and Dunn's multiple comparisons test. Source data are provided as a Source Data file. Presentation of full scan blots are provided in the Source data file. For quantification blots were processed in parallel.

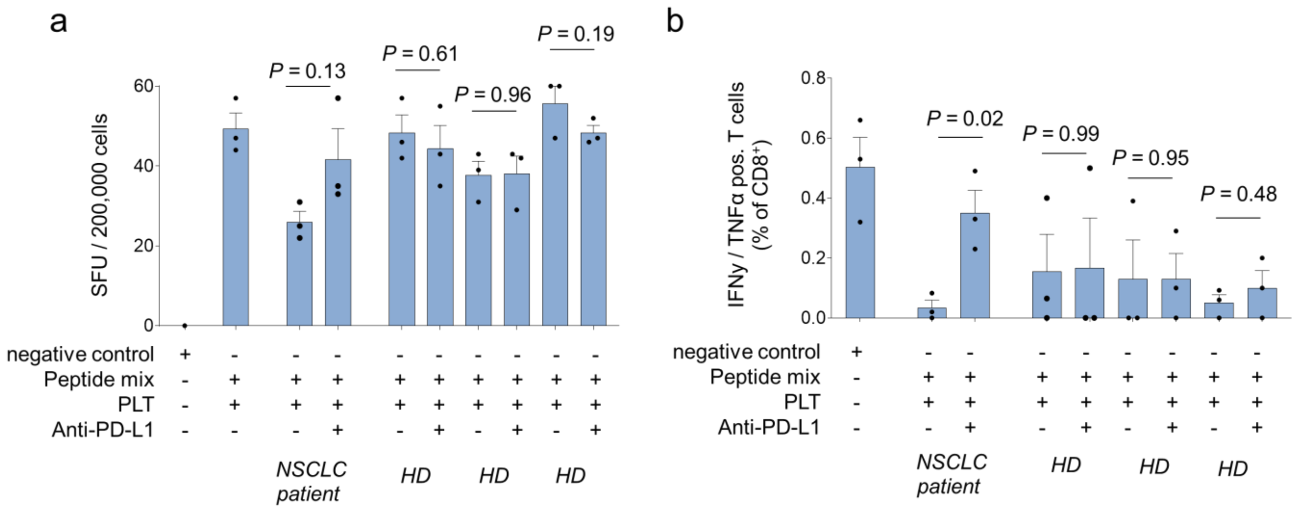

Supplementary Figure 6: Immunomodulation of platelets derived from healthy donors. a Quantification of the IFNγ ELISPOT assays in three healthy donors and one NSCLC patient. b Flow cytometry-based quantification of indicated cytokines and surface markers for peptide stimulated CD8+ T-cells co-incubated with PD-L1 positive platelets with or without anti-PD-L1 mAb pre-treatment (n = 4). a-b Data are mean ± SEM. Statistical significance was calculated by two-tailed Student's t-test. Source data are provided as a Source Data file.

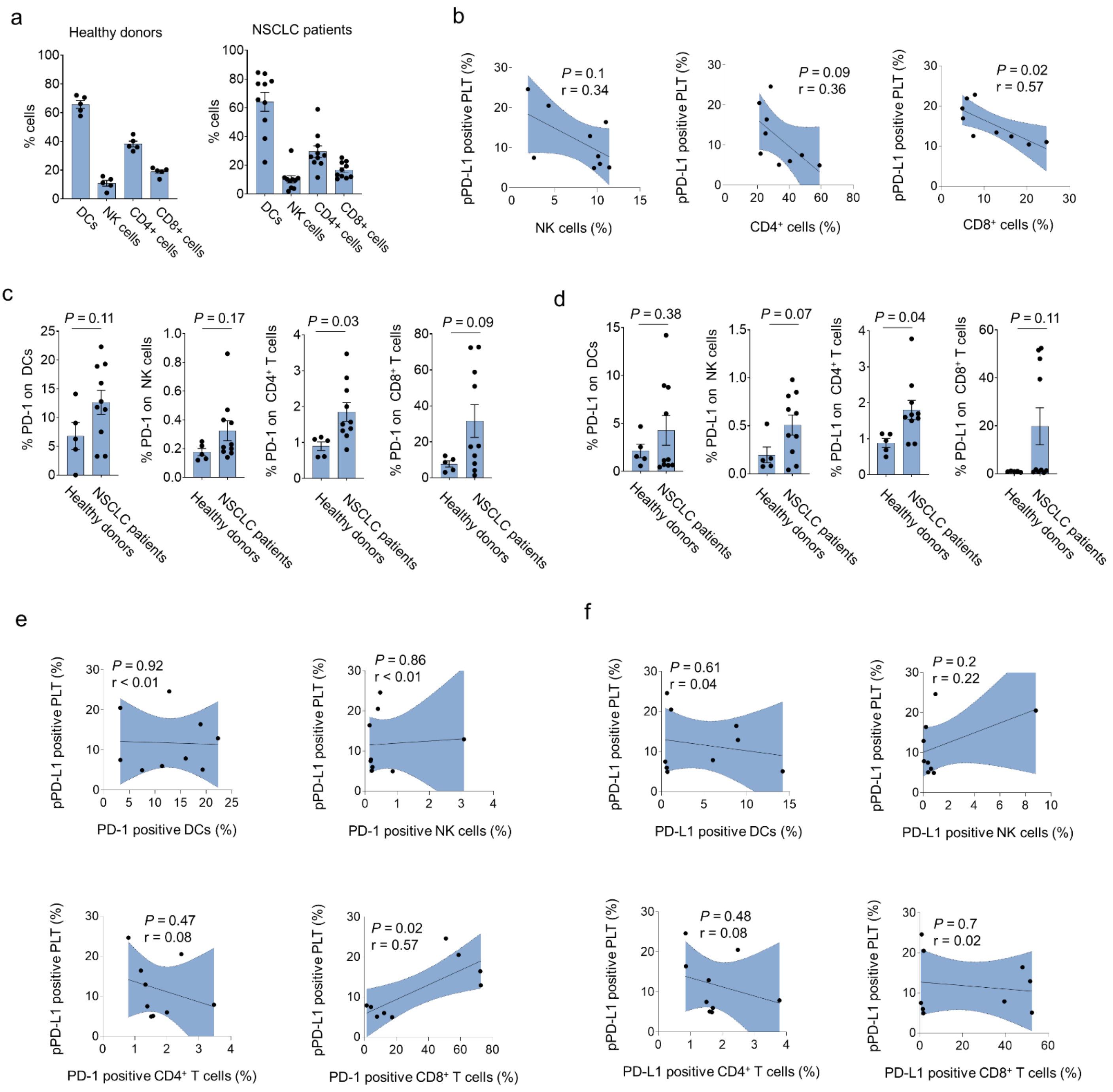

Supplementary Figure 7: Immunophenotyping in NSCLC patients. a Flow cytometry-based determination of immune cell distribution in the peripheral blood of healthy donors (n = 5) and NSCLC patients (n = 10). Data are mean ± SEM. **b** Correlation of pPD-L1 level and total number of NK, $CD4^+$ and $CD8^+$ cells. Correlation was determined by simple linear regression analysis. (n = 9) **c** Analysis of PD-1 expression on DCs, NK cells $CD4^+$ and $CD8^+$ cells in healthy donors and NSCLC patients. **d** Analysis of PD-L1 expression (%) on DCs, NK cells $CD4^+$ and $CD8^+$ cells in healthy donors and NSCLC patients. **c-d** Data are mean ± SEM. Statistical significance was calculated by two-tailed Student's *t*-test (c left, c middle right, d middle right) and two-tailed Mann-Whitney test (c middle left, c right, d left, d middle left, d left). **e** Correlation of pPD-L1 level and number of PD-1 positive NK, $CD4^+$ and $CD8^+$ cells. Correlation was determined by simple linear regression analysis (n = 9). **f** Correlation of pPD-L1 level and number of PD-L1 positive NK, $CD4^+$ and $CD8^+$ cells (n = 9). Correlation was determined by simple linear regression analysis. Source data are provided as a Source Data file. The gating strategy applied for the evaluation of flow-cytometry-acquired data presented in this figure is provided in Supplementary Fig. 13.

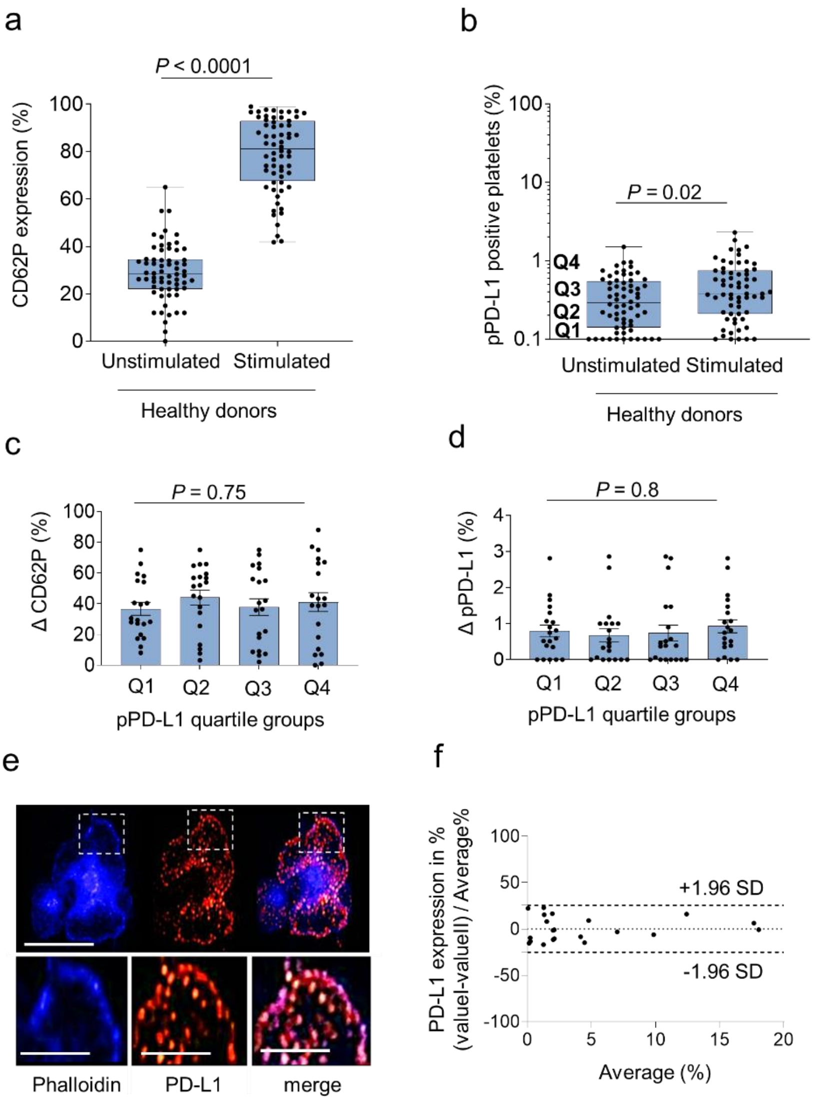

Supplementary Figure 8: Association of pPD-L1 expression and platelet activation in healthy donors. a-b Changes in CD62P (a) and the PD-L1 (b) levels upon platelet stimulation with 10µM TRAP-6 for 2 minutes in 64 healthy donors. Statistical significance was calculated by two-tailed Mann Whitney test. Boxes represent median and 25th to 75th percentiles, whiskers are minimum to maximum. c CD62 expression change (ΔCD62P) in the different pPD-L1 quartile groups identified in unstimulated platelets of healthy donors (n = 20). d PD-L1 expression change (ΔPD-L1) in the different pPD-L1 quartile groups identified in unstimulated platelets of healthy donors (n = 20). Data are mean ± SEM. Statistical significance was calculated by Kruskal-Wallis test and Dunn's multiple comparisons test. e Representative PD-L1 immunofluorescent staining on platelets derived from a NSCLC patient (TP123). Lower section shows a detailed expression pattern the PD-L1 on platelets. Platelets were counter stained with phalloidin. Up, scale bar 2 µm, lower scale bar 0.5 µm, (n = 1). f Bland-Altman plot for the flow cytometry-based determination of PD-L1 on the platelet surface (big dotted line displays: -1.96SD: -25.17, +1.96SD: 25.5, Bias: 0.17) (n = 21). Source data are provided as a Source Data file.

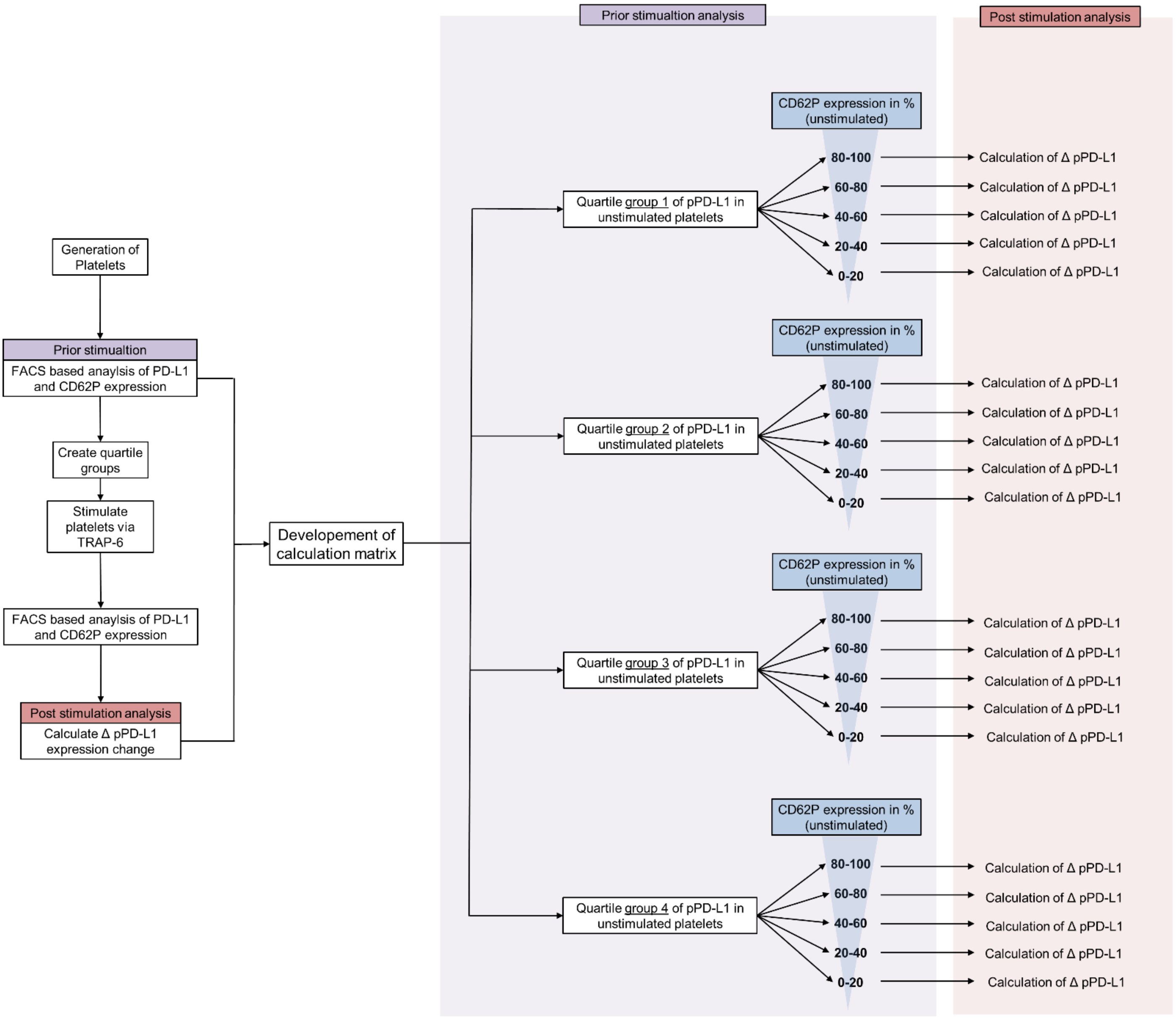

Supplementary Figure 9: Algorithm of pPD-L1Adj calculation. Flow chart presenting the establishment of an activation-independent calculation matrix for platelet PD-L1.

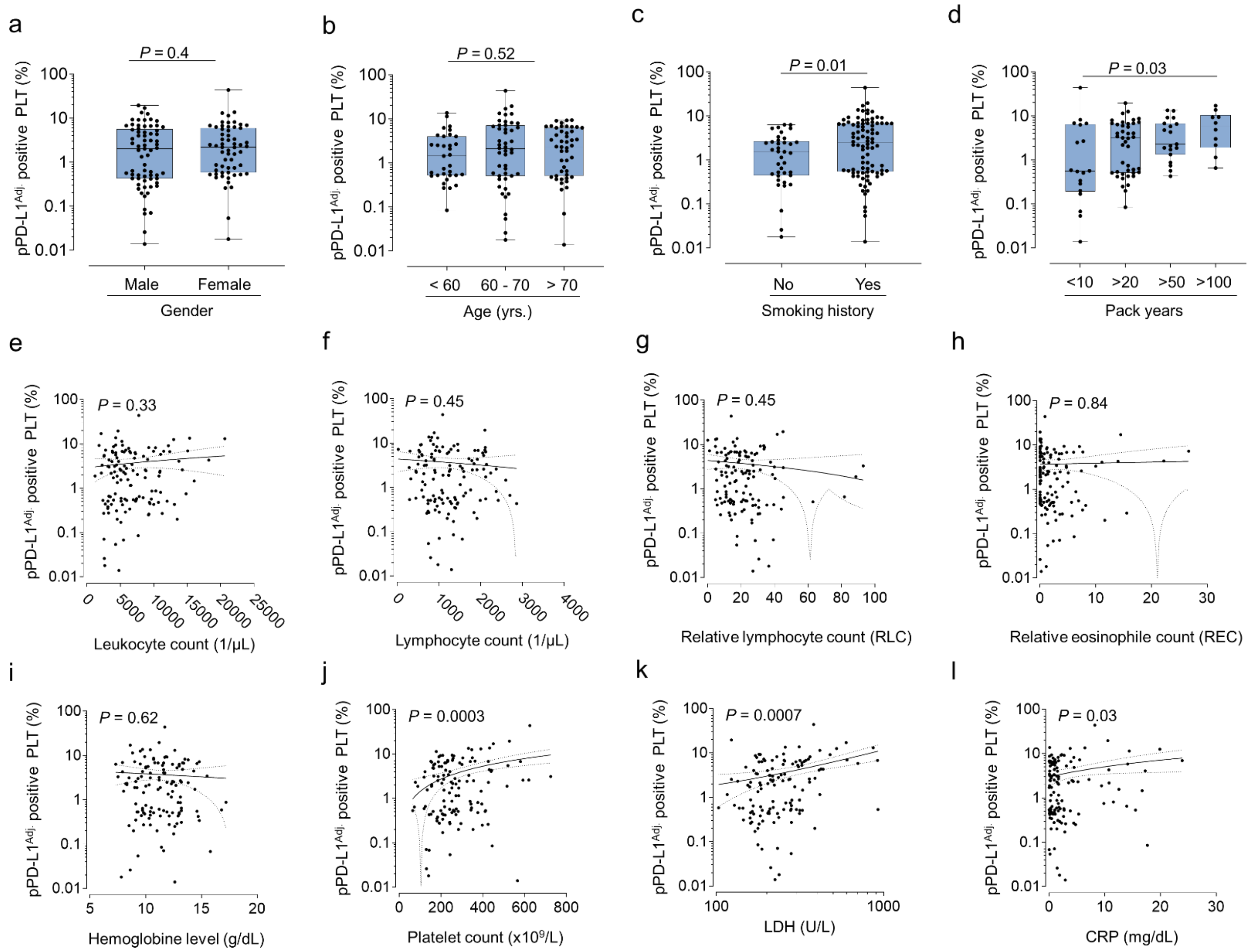

Supplementary Figure 10: Correlation of pPD-L1Adj and clinical parameters. a Correlation of pPD-L1Adj levels and gender (n = 128, male = 69, female = 59). Each dot represents a single patient. Data are median and IQR. Statistical significance was calculated by two-tailed Mann-Whitney test. b Correlation of pPD-L1Adj levels and age (n = 128, < 60 = 33, 60-70 = 48, >70 = 47). Each dot represents a single patient. Data are median and IQR. Statistical significance was calculated by Kruskal-Wallis test. c Correlation of pPD-L1Adj levels and smoking history (n = 128, no = 38, yes = 90). Each dot represents a single patient. Data are median and IQR. Statistical significance was calculated by two-tailed Mann-Whitney test. d Correlation of pPD-L1Adj levels and pack years (n = 90, <10 = 18, >20 = 43, >50 = 19, >100 = 10). Each dot represents a single patient. Statistical significance was calculated by Kruskal-Wallis test. a-d Boxes represent median and 25th to 75th percentiles, whiskers are minimum to maximum. e-l Correlation of pPD-L1Adj levels and leukocyte count (n = 128) (e), lymphocyte count (n = 127) (f), relative lymphocyte count (n = 128) (g), relative eosinophil count (n = 128) (h), haemoglobin level (n = 128) (i), platelet count (n = 128) (j), LDH (n = 128) (k) and CRP (n = 122) (l). Each dot represents a single patient. Correlation was determined by simple linear regression analysis. Source data are provided as a Source Data file.

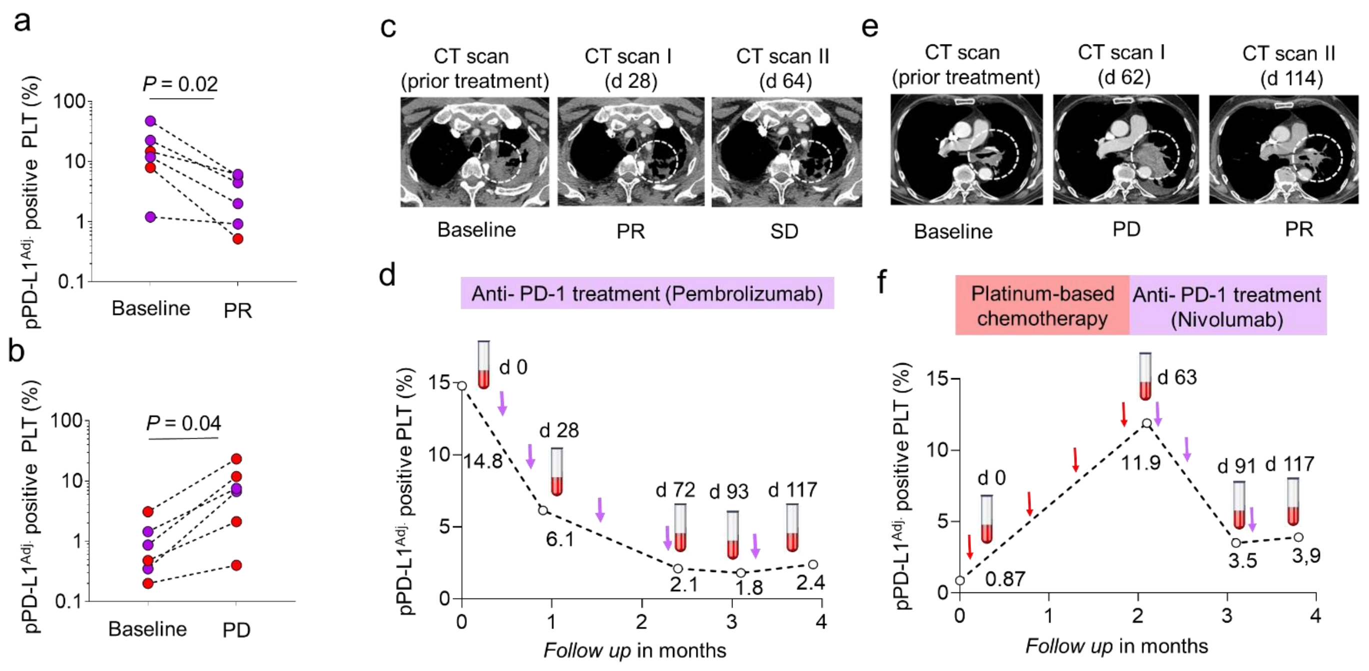

Supplementary Figure 11: pPD-L1 predicts treatment response in NSCLC. a-b pPD-L1Adj levels differ in patients presenting with a partial remission (PR) or a progressive disease (PD) (n = 6). Purple dots represent anti-PD-1 treatment, red dots platinum-based chemotherapy. Statistical significance was calculated by two-tailed Mann-Whitney test. c-f Long-term pPD-L1Adj profiles of two representative NSCLC patients (arrow represents treatment, dots represent determination of pPD-L1Adj). Source data are provided as a Source Data file.

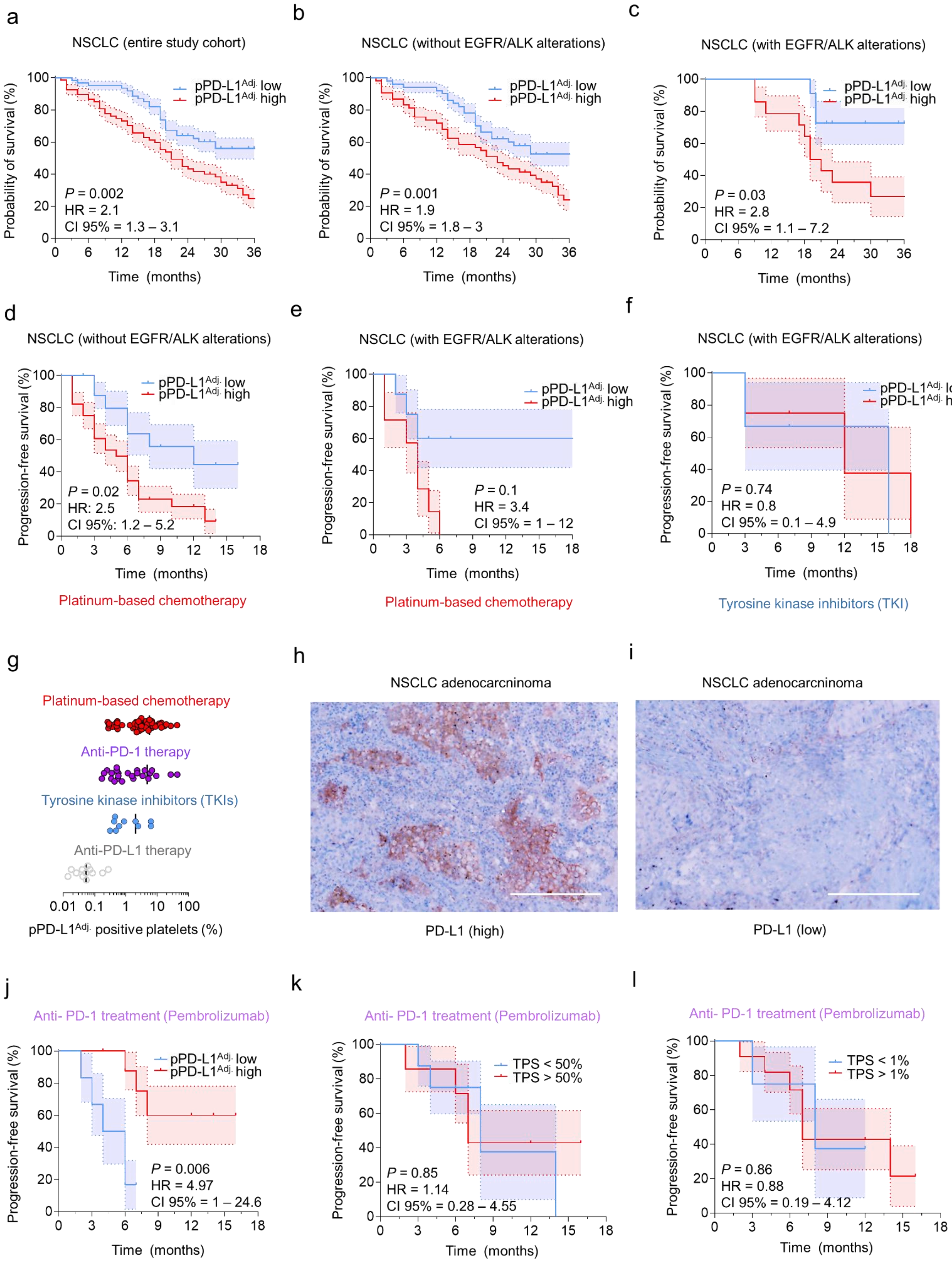

a
NSCLC (entire study cohort)
pPD-L1Adj. low
pPD-L1Adj. high
P = 0.002
HR = 2.1
CI 95% = 1.3 – 3.1
Probability of survival (%)
Time (months)
b
NSCLC (without EGFR/ALK alterations)
P = 0.001
HR = 1.9
CI 95% = 1.8 – 3
c
NSCLC (with EGFR/ALK alterations)
P = 0.03
HR = 2.8
CI 95% = 1.1 – 7.2
d
NSCLC (without EGFR/ALK alterations)
P = 0.02
HR: 2.5
CI 95%: 1.2 – 5.2
Progression-free survival (%)
Platinum-based chemotherapy
e
NSCLC (with EGFR/ALK alterations)
P = 0.1
HR = 3.4
CI 95% = 1 – 12
Platinum-based chemotherapy
f
NSCLC (with EGFR/ALK alterations)
P = 0.74
HR = 0.8
CI 95% = 0.1 – 4.9
Tyrosine kinase inhibitors (TKI)
g
Platinum-based chemotherapy
Anti-PD-1 therapy
Tyrosine kinase inhibitors (TKIs)
Anti-PD-L1 therapy
pPD-L1Adj. positive platelets (%)
h
NSCLC adenocarcninoma
PD-L1 (high)
i
NSCLC adenocarcninoma
PD-L1 (low)
j
Anti- PD-1 treatment (Pembrolizumab)
P = 0.006
HR = 4.97
CI 95% = 1 – 24.6
k
Anti- PD-1 treatment (Pembrolizumab)
TPS < 50%
TPS > 50%
P = 0.85
HR = 1.14
CI 95% = 0.28 – 4.55
l
Anti- PD-1 treatment (Pembrolizumab)
TPS < 1%
TPS > 1%
P = 0.86
HR = 0.88
CI 95% = 0.19 – 4.12

Supplementary Figure 12: pPD-L1 as prognostic and predictive marker in NSCLC. a Kaplan–Meier curves estimates of OS in the entire study cohort (n = 128, high = 67, low = 61). b-c Kaplan–Meier curves estimates of OS in NSCLC patients with (b, n = 103, high = 53, low = 50) or without EGFR/ALK alterations (c, n = 26, high = 15, low = 11).. d Kaplan–Meier curves estimates of PFS in NSCLC patients without EGFR/ALK alterations receiving platinum-based chemotherapy (n = 47, high = 30, low = 17).. e Kaplan–Meier curves estimates of PFS in NSCLC patients with EGFR/ALK alterations receiving platinum-based chemotherapy (n = 15, high = 7, low = 8).. f Kaplan–Meier curves estimates of PFS in NSCLC patients with EGFR/ALK alterations receiving tyrosine kinase inhibitors (TKI) (n = 7, high = 4, low = 3).. a-f Statistical significance was calculated by log-rank test, pPD-L1Adj level > median (red) and pPD-L1Adj level < median (blue). g pPD-L1Adj expression regarding to the treatment regimens. h-i Representative immunohistochemistry showing PD-L1 expression in two NSCLC patients. Scale bar 250 µm, (n = 2). j Kaplan–Meier curves estimates of PFS in NSCLC patients receiving the anti-PD-1 treatment (Pembrolizumab) (n = 15, high = 9, low = 6). pPD-L1Adj level > median (red) and pPD-L1Adj level < median (blue). k-l Kaplan–Meier curves estimates of PFS in NSCLC patients receiving the anti-PD-1 treatment (Pembrolizumab) (n = 15). TPS >50% (k, n = 7) and TPS>1% (l, n = 11) is given in red, TPS< 50% (k, n = 8) and TPS < 1% (l, n = 4) is given in blue. j -l Statistical significance was calculated by log-rank test. Source data are provided as a Source Data file.

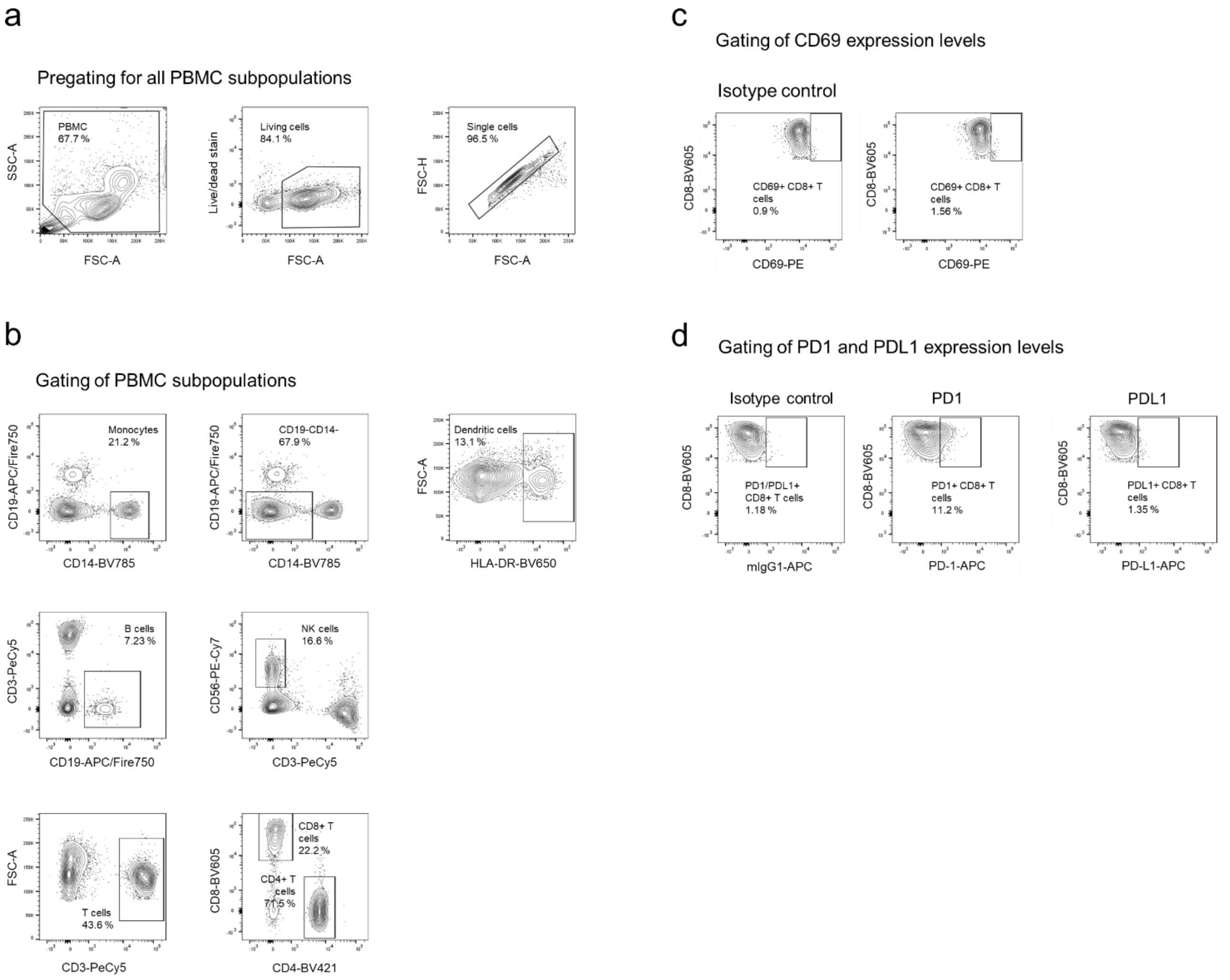

Supplementary Figure 13: Exemplary FACS gating strategies. a FACS gating strategy for pre-gating of PBMC subpopulations. b FACS gating strategy for identification of PBMC subpopulations. c FACS gating strategy for determination of CD69 expression. d FACS gating strategy for determination of PD-1 and PD-L1 expression.

Supplementary Table 1: Patients characteristics of the proof of principle (PoP) cohort

Patient characteristics	Total
**Gender**	
male sex, n (%)	71 (55.5)
**Age**	
Age at study inclusion in years, mean–yr.(95 % CI)	65.7 ± 14.3 (19 to 87)
**Histopathological subtype, n (%)**	
Adenocarcinoma	91 (71.1)
Squamous cell carcinoma	35 (27.3)
Large cell carcinoma	2 (1.6)
Unspecified	0
**TNM classification, n (%)**	
Stage	
Tx	15 (11.7)
T1	11 (8.6)
T2	30 (23.4)
T3	24 (18.8)
T4	48 (37.5)
Node	
Nx	9 (7)
N0	11 (8.6)
N1	13 (10.2)
N2	39 (21.7)
N3	56 (43.8)
Metastasis	
M0	22 (17.2)
M1	106 (82.8)
**UICC stage, n (%)**	
I	1 (0.8)
II	6 (4.7)
III	13 (10.2)
IV	108 (84.4)
**Localization in lung on CT, n (%)**	

Central	43 (33.6)
Peripheral	71 (55.5)
Not available	14 (10.9)
**Smoking status, n (%)**	
Current or former smoker	96 (75)
Never smoked	14 (10.9)
Unknown	18 (14.1)
**Genetic EGFR aberration , n (%)**	19 (14.8)
**Genetic ALK aberration , n (%)**	5 (3.9)
**Genetic ROS aberration , n (%)**	6 (4.7)
**Genetic KRAS aberration , n (%)**	38 (29.7)
**Number of prior systemic therapy, n (%)**	
1	72 (56.3)
2	31 (24.2)
≥3	25 (19.5)
**Type of prior therapy, n (%)**	
Surgery	23 (17.9)
Radiation	4 (1.8)
Chemotherapy	104 (47.3)
Tyrosine kinase inhibitor	30 (13.6)
Anti-PD-1/PD-L1 therapy	75 (34.1)

n=number, yr.=year, %=percentage, T=tumor, N=lymph node, M=metastasis, x=undefined, UICC=Union for International Cancer Control, EGRF= Epidermal Growth Factor Receptor

Supplementary Table 2: Antibodies

Antibody	Clone	Fluorochrome	Vendor	Catalogue number	Dilution
Anti-CD41a	REA386	VB	Miltenyi Biotec	130-105-561	1:25 Flow cytometry
Anti-CD41a	HIP8	PeCy5	BioLegend	303708	1:25 Flow cytometry
Anti-CD62P	AK-4	FITC	ThermoFisher	11-0628-42	1:20 Flow cytometry
Anti-PD-L1	MIH-1	APC	ThermoFisher	17-5983-42	1:50 Flow cytometry
REA control	REA293	VB	Miltenyi Biotec	130-113-438	1:50 Flow cytometry
Anti-Human IgG	-	APC	abcam	99768	1:50 Flow cytometry
Anti-Human IgG	-	FITC	abcam	99772	1:20 Flow cytometry
Anti.Human IgG	-	PeCy5	BD	551497	1:25 Flow cytometry
Anti-PD-L1	28-8	-	Abcam	205921	1:250 IF/PLA
Anti-CD61	SJ-19-09	-	ThermoFisher	32077	1:1000 IF
Anti-CD3	Okt 03	BV-510	BioLegend	300448	1:200 Flow cytometry
Anti-CD56	HCD56	BV605	BioLegend	318334	1:200 Flow cytometry
Anti-CD45RO	HI100	BV785	BioLegend	304234	1:200 Flow cytometry
Anti-CD4	RPA-T4	APC-Cy7	BioLegend	300518	1:100 Flow cytometry
Anti-CD8	SFCI21Thy2D3	PE-Cy7	Beckman Coulter	737661	1:400 Flow cytometry
Anti-CD27	M-T271	PE-CF594	BD Biosience	562324	1:200 Flow cytometry
Anti-CD28	CD28.2	PE-Cy7	BioLegend	302926	1:200 Flow cytometry
Anti-CD62L	DREG-56	FITC	BioLegend	304810	1:400 Flow cytometry
Anti-GFP	EPR14104	-	Abcam	183734	1:500 IF
Anti-IFNy	4SB3	APC	BioLegend	502512	1:200 Flow cytometry
Anti-TNFα	Mab11	Pacific blue	BioLegend	502920	1:120 Flow cytometry

Anti-GFP	EPR14104	-	Abcam	183734	1:500 IF
Anti-Fibronectin	P1H11	-	Novus Biologicals	MAB1918	1:200 IF/PLA
Anti-CD3	UCHT-1	PeCy5	BD	555334	1:25 Flow cytometry
Anti-CD19	HIB19	APC/Fire750	Biolegend	302258	1:100 Flow cytometry
Anti-CD4	RPA-T4	Brilliant Violet 421	Biolegend	300532	1:100 Flow cytometry
Anti-CD8	RPA-T8	BV605	Biolegend	301040	1:100 Flow cytometry
Anti-CD16	CB16	FITC	ThermoFisher	11-0168-42	1:50 Flow cytometry
Anti-CD56	5.1H11	PE-Cy7	Biolegend	362510	1:50 Flow cytometry
Anti-CD14	M5E2	BV785	Biolegend	301840	1:100 Flow cytometry
Anti-HLA-DR	L243	BV650	Biolegend	307650	1:100 Flow cytometry
Anti-PD-1	EH12.2H7	APC	Biolegend	329908	1:100 Flow cytometry
Anti-PD-L1	MIH2	APC	Biolegend	393610	1:100 Flow cytometry
Anti-CD69	FN50	PE	BD	557050	1:60 Flow cytometry
Anti-mIgG1	MOPC21	PE	BD	555749	1:60 Flow cytometry
Anti-mIgG1	MOPC21	APC	BD	550854	1:60 Flow cytometry
Anti-Cytokeratin	REA831	PE	Miltenyi Biotec	130-112-744	1:50 MACSima
Anti-CD2	REA1130	PE	Miltenyi Biotec	130-119-508	1:50 MACSima
Anti-CD279	REA1165	PE	Miltenyi Biotec	130-120-382	1:50 MACSima
Anti-CD3	REA1151	FITC	Miltenyi Biotec	130-120-267	1:50 MACSima
