# Supplementary material for: Platelet PD-L1 reflects collective intratumoral PD-L1 expression and predicts immunotherapy response in non-small cell lung cancer
Source: Nat Commun. 2021 Dec 1;12:7005. doi: 10.1038/s41467-021-27303-7 (PMC8636618; doi:10.1038/s41467-021-27303-7)
Supplement: Supplementary file 2 — Reporting Summary [file 41467_2021_27303_MOESM2_ESM.pdf]

nature portfolio

Corresponding author(s): Lars Zender
Last updated by author(s): Oct 28, 2021

Reporting Summary

Nature Portfolio wishes to improve the reproducibility of the work that we publish. This form provides structure for consistency and transparency in reporting. For further information on Nature Portfolio policies, see our Editorial Policies and the Editorial Policy Checklist.

Statistics

For all statistical analyses, confirm that the following items are present in the figure legend, table legend, main text, or Methods section.

n/a	Confirmed	
☐	☒	The exact sample size (*n*) for each experimental group/condition, given as a discrete number and unit of measurement
☐	☒	A statement on whether measurements were taken from distinct samples or whether the same sample was measured repeatedly
☐	☒	The statistical test(s) used AND whether they are one- or two-sided<br>*Only common tests should be described solely by name; describe more complex techniques in the Methods section.*
☐	☒	A description of all covariates tested
☐	☒	A description of any assumptions or corrections, such as tests of normality and adjustment for multiple comparisons
☐	☒	A full description of the statistical parameters including central tendency (e.g. means) or other basic estimates (e.g. regression coefficient) AND variation (e.g. standard deviation) or associated estimates of uncertainty (e.g. confidence intervals)
☐	☒	For null hypothesis testing, the test statistic (e.g. *F*, *t*, *r*) with confidence intervals, effect sizes, degrees of freedom and *P* value noted<br>*Give P values as exact values whenever suitable.*
☒	☐	For Bayesian analysis, information on the choice of priors and Markov chain Monte Carlo settings
☒	☐	For hierarchical and complex designs, identification of the appropriate level for tests and full reporting of outcomes
☐	☒	Estimates of effect sizes (e.g. Cohen's *d*, Pearson's *r*), indicating how they were calculated

Our web collection on statistics for biologists contains articles on many of the points above.

Software and code

Policy information about availability of computer code

Data collection	FACS canto II cytometer, CellSens Dimension 1.17, ImageLab v5.2.1, 7500 Software v2.0.6, ImmunoSpot S5 analyzer
Data analysis	GraphPad Prism (v.8.1.0, v.8.4.0), ImageJ (v1.51n, v.1.52), BioRender software (BioRender.com), SPSS (v21, v27) , 7500 Software v2.0.6, ApEv.2.0.51, FlowJo software version 10.0.8

For manuscripts utilizing custom algorithms or software that are central to the research but not yet described in published literature, software must be made available to editors and reviewers. We strongly encourage code deposition in a community repository (e.g. GitHub). See the Nature Portfolio guidelines for submitting code & software for further information.

Data

Policy information about availability of data

All manuscripts must include a data availability statement. This statement should provide the following information, where applicable:

- Accession codes, unique identifiers, or web links for publicly available datasets
- A description of any restrictions on data availability
- For clinical datasets or third party data, please ensure that the statement adheres to our policy

All data generated in this study are available within the Article, Supplementary Information or Source Data file. Source data are provided with this paper.

Field-specific reporting

Please select the one below that is the best fit for your research. If you are not sure, read the appropriate sections before making your selection.

[x] Life sciences [ ] Behavioural & social sciences [ ] Ecological, evolutionary & environmental sciences

For a reference copy of the document with all sections, see nature.com/documents/nr-reporting-summary-flat.pdf

Life sciences study design

All studies must disclose on these points even when the disclosure is negative.

Sample size	This was a discovery project for identifiying and investigating the predicitve and prognostic role of PD-L1 expression on platelets in non-small cell lung cancer (NSCLC). Since there are no valid data existing on the expression of PDL-1 on platelets in a NSCLC cohort, we assumed platelet PDL-1 detection in approximately 5-10% of cells. The statistical estimation of the total number of patients required was performed regarding: $n=(u+v)^2(ó1^2+ ó^2)/("1-"0)^2$ and estimated > 100. A total of n = 173 NSCLC patients (screening cohort =SC) and n = 65 healthy donors were included. Out of the SC, n = 5 patients with loss of follow up were excluded. The resulting study cohort included n = 168. In addition n = 40 patients on on LMWH, aspirin and/or thienopyridines (e,g. clopidogrel) or other oral anticoagulants were excluded from final analyses. The resulting proof of principle cohort (PoP) consists n = 128. In addition n = 14 NSCLC were analyzed repetitively.
Data exclusions	As stated above, out of the primary SC, n=5 patients were excluded due to loss of follow up. In addition, n =40 patients taking mediactions affecting platelet function and/or coagulation were excluded in the PoP.
Replication	In order to verify the reproducibility of our flow cytometry system for determination of platelet-expressed PD-L1 (pPD-L1), we performed a Bland–Altman analysis in n =21 NSCLC samples. Elispot analysis were assessed as duplicates and only reliable replicates were analyzed.
Randomization	No randomization was performed. Since this study aims to explore and reveal the role of PD-L1 expression on platelets in a real live cohort of non-small cell lung cancer (NSCLC) patients, no specific randomisation into different NSCLC subgroups was done.
Blinding	Since no concomitant therapeutic intervention was performed, blinding was not appropriate for this study. Of note, recruiting physicians were not involved in sample preparation and consecutive analysis. Clincial data analysis were performed from operators not involved in the recruiting process. Data analysis was performed with anonymized patient data at different time points after patient recruitment. Thus, the operator had no influence on recruitment and was not aware of the clinical course. Most in vitro experiments were carried out and analyzed by two different operators.

Reporting for specific materials, systems and methods

We require information from authors about some types of materials, experimental systems and methods used in many studies. Here, indicate whether each material, system or method listed is relevant to your study. If you are not sure if a list item applies to your research, read the appropriate section before selecting a response.

Materials & experimental systems

n/a	Involved in the study	
[ ]	[x]	Antibodies
[ ]	[x]	Eukaryotic cell lines
[x]	[ ]	Palaeontology and archaeology
[x]	[ ]	Animals and other organisms
[ ]	[x]	Human research participants
[x]	[ ]	Clinical data
[x]	[ ]	Dual use research of concern

Methods

n/a	Involved in the study	
[x]	[ ]	ChIP-seq
[ ]	[x]	Flow cytometry
[x]	[ ]	MRI-based neuroimaging

Antibodies

Antibodies used	Anti-CD41a clone: REA386 fluorchrome: VB vendor: Miltenyi Biotec Catalogue number: 130-105-561 1:25 Flow cytometry<br>Anti-CD41a clone: HIP8 PeCy5 vendor: BioLegend Catalogue number: 303708 1:25 Flow cytometry<br>Anti-CD62P clone: AK-4 FITC vendor: ThermoFisher Catalogue number: 11-0628-42 1:20 Flow cytometry<br>Anti-PD-L1 clone: MIH-1 APC vendor: ThermoFisher Catalogue number: 17-5983-42 1:50 Flow cytometry<br>REA control clone: REA293 VB vendor: Miltenyi Biotec Catalogue number: 130-113-438 1:50 Flow cytometry<br>Anti-Human IgG clone: - APC vendor: abcam Catalogue number: 99768 1:50 Flow cytometry<br>Anti-Human IgG clone: - FITC vendor: abcam Catalogue number: 99772 1:20 Flow cytometry<br>Anti.Human IgG clone: - PeCy5 vendor: BD Catalogue number: 551497 1:25 Flow cytometry<br>Anti-PD-L1 clone: 28-8 - vendor: Abcam Catalogue number: 205921 1:250 IF/PLA<br>Anti-CD61 clone: SJ-19-09 - vendor: ThermoFisher Catalogue number: 32077 1:1000 IF<br>Anti-CD3 clone: Okt 03 BV-510 vendor: BioLegend Catalogue number: 300448 1:200 Flow cytometry

Anti-CD56 clone: HCD56 BV605 vendor: BioLegend Catalogue number: 318334 1:200 Flow cytometry
Anti-CD45RO clone: HI100 BV785 vendor: BioLegend Catalogue number: 304234 1:200 Flow cytometry
Anti-CD4 clone: RPA-T4 APC-Cy7 vendor: BioLegend Catalogue number: 300518 1:100 Flow cytometry
Anti-CD8 clone: SFCI21Thy2D3 PE-Cy7 vendor: Beckman Coulter Catalogue number: 737661 1:400 Flow cytometry
Anti-CD27 clone: M-T271 PE-CF594 vendor: BD Biosience Catalogue number: 562324 1:200 Flow cytometry
Anti-CD28 clone: CD28.2 PE-Cy7 vendor: BioLegend Catalogue number: 302926 1:200 Flow cytometry
Anti-CD62L clone: DREG-56 FITC vendor: BioLegend Catalogue number: 304810 1:400 Flow cytometry
Anti-GFP clone: EPR14104 - vendor: Abcam Catalogue number: 183734 1:500 IF
Anti-IFNy clone: 4SB3 APC vendor: BioLegend Catalogue number: 502512 1:200 Flow cytometry
Anti-TNFα clone: Mab11 Pacific blue vendor: BioLegend Catalogue number: 502920 1:120 Flow cytometry
Anti-GFP clone: EPR14104 - vendor: Abcam Catalogue number: 183734 1:500 IF
Anti-Fibronectin clone: P1H11 - vendor: Novus Biologicals Catalogue number: MAB1918 1:200 IF/PLA
Anti-CD3 clone: UCHT-1 PeCy5 vendor: BD Catalogue number: 555334 1:25 Flow cytometry
Anti-CD19 clone: HIB19 APC/Fire750 vendor: Biolegend Catalogue number: 302258 1:100 Flow cytometry
Anti-CD4 clone: RPA-T4 Brilliant Violet 421 vendor: Biolegend Catalogue number: 300532 1:100 Flow cytometry
Anti-CD8 clone: RPA-T8 BV605 vendor: Biolegend Catalogue number: 301040 1:100 Flow cytometry
Anti-CD16 clone: CB16 FITC vendor: ThermoFisher Catalogue number: 11-0168-42 1:50 Flow cytometry
Anti-CD56 clone: 5.1H11 PE-Cy7 vendor: Biolegend Catalogue number: 362510 1:50 Flow cytometry
Anti-CD14 clone: M5E2 BV785 vendor: Biolegend Catalogue number: 301840 1:100 Flow cytometry
Anti-HLA-DR clone: L243 BV650 vendor: Biolegend Catalogue number: 307650 1:100 Flow cytometry
Anti-PD-1 clone: EH12.2H7 APC vendor: Biolegend Catalogue number: 329908 1:100 Flow cytometry
Anti-PD-L1 clone: MIH2 APC vendor: Biolegend Catalogue number: 393610 1:100 Flow cytometry
Anti-CD69 clone: FN50 PE vendor: BD Catalogue number: 557050 1:60 Flow cytometry
Anti-mIgG1 clone: MOPC21 PE vendor: BD Catalogue number: 555749 1:60 Flow cytometry
Anti-mIgG1 clone: MOPC21 APC vendor: BD Catalogue number: 550854 1:60 Flow cytometry
Anti-Cytokeratin clone: REA831 PE vendor: Miltenyi Biotec Catalogue number: 130-112-744 1:50 MACSima
Anti-CD2 clone: REA1130 PE vendor: Miltenyi Biotec Catalogue number: 130-119-508 1:50 MACSima
Anti-CD279 clone: REA1165 PE vendor: Miltenyi Biotec Catalogue number: 130-120-382 1:50 MACSima
Anti-CD3 clone: REA1151 FITC vendor: Miltenyi Biotec Catalogue number: 130-120-267 1:50 MACSima

Validation

We provided a link for the relevant information and data sheet for each antibody. The data sheet includes the validation of all primary antibodies for the species and application, quality control procedures and relevant citations:
Anti-CD41a https://www.miltenyibiotec.com/LU-en/products/cd41a-antibody-anti-human-reafinity-rea386.html#apc:100-tests-in-1-ml
Anti-CD41a https://www.biolegend.com/en-us/products/pe-cyanine5-anti-human-cd41-antibody-738?GroupID=BLG5923
Anti-CD62P https://www.thermofisher.com/antibody/product/CD62P-P-Selectin-Antibody-clone-AK-4-Monoclonal/11-0628-42
Anti-PD-L1 https://www.thermofisher.com/antibody/product/CD274-PD-L1-B7-H1-Antibody-clone-MIH1-Monoclonal/17-5983-42
REA control https://www.miltenyibiotec.com/LU-en/products/rea-control-antibody-s-human-igg1-reafinity-rea293.html#pe:100-tests-in-200-ul
Anti-Human IgG https://www.abcam.com/mouse-monoclonal-h2-human-igg-fc-apc-ab99768.html
Anti-Human IgG https://www.abcam.com/mouse-monoclonal-4e3-human-igg1-hinge-heavy-chain-fitc-ab99772.html
Anti.Human IgG https://www.bdbiosciences.com/en-us/products/reagents/flow-cytometry-reagents/research-reagents/single-color-antibodies-ruo/pe-cy-5-mouse-anti-human-igg.551497
Anti-PD-L1 https://www.abcam.com/pd-l1-antibody-28-8-ab205921.html
Anti-CD61 https://www.thermofisher.com/antibody/product/CD61-Integrin-beta-3-Antibody-clone-SJ19-09-Recombinant-Monoclonal/MA5-32077
Anti-CD3 https://www.biolegend.com/en-us/products/brilliant-violet-510-anti-human-cd3-antibody-9792?GroupID=BLG5900
Anti-CD56 https://www.biolegend.com/en-us/products/brilliant-violet-605-anti-human-cd56-ncam-antibody-7668?GroupID=BLG15664
Anti-CD45RO https://www.biolegend.com/de-de/products/brilliant-violet-785-anti-human-cd45ro-antibody-7973?GroupID=GROUP658
Anti-CD4 https://www.biolegend.com/en-us/products/apc-cyanine7-anti-human-cd4-antibody-1933?GroupID=BLG5901
Anti-CD8 https://www.beckman.de/reagents/coulter-flow-cytometry/antibodies-and-kits/single-color-antibodies/cd8/737661
Anti-CD27 https://www.fishersci.de/shop/products/anti-cd27-pe-cf594-clone-m-t271-bd/15861819
Anti-CD28 https://www.biolegend.com/en-us/search-results/pe-cyanine7-anti-human-cd28-antibody-6773
Anti-CD62L https://www.biolegend.com/en-us/search-results/apc-anti-human-cd62l-antibody-650?gclid=EAIaIQobChMIgr7E3Obq8wIVgbh3Ch2MKAaiEAAYAiAAEgKqAfD_BwE
Anti-GFP https://www.abcam.com/gfp-antibody-epr14104-ab183734.html
Anti-IFNy https://www.biolegend.com/en-us/products/apc-anti-human-ifn-gamma-antibody-1012
Anti-TNFα https://www.biolegend.com/en-us/search-results/pacific-blue-anti-human-tnf-alpha-antibody-4149
Anti-GFP https://www.abcam.com/gfp-antibody-epr14104-ab183734.html
Anti-Fibronectin https://www.novusbio.com/products/fibronectin-antibody-p1h11_mab1918
Anti-CD3 https://www.bdbiosciences.com/en-ca/products/reagents/flow-cytometry-reagents/research-reagents/single-color-antibodies-ruo/pe-cy-5-mouse-anti-human-cd3.555334
Anti-CD19 https://www.biolegend.com/en-us/search-results/apc-fire-750-anti-human-cd19-antibody-13564
Anti-CD4 https://www.biolegend.com/en-us/search-results/brilliant-violet-421-anti-human-cd4-antibody-7151
Anti-CD8 https://www.biolegend.com/en-us/products/brilliant-violet-605-anti-human-cd8a-antibody-7651
Anti-CD16 https://www.thermofisher.com/antibody/product/CD16-Antibody-clone-eBioCB16-CB16-Monoclonal/11-0168-42

Anti-CD56 https://www.biolegend.com/en-us/products/pe-cyanine7-anti-human-cd56-ncam-antibody-9959
Anti-CD14 https://www.biolegend.com/en-us/products/brilliant-violet-785-anti-human-cd14-antibody-7965
Anti-HLA-DR https://www.biolegend.com/en-us/products/brilliant-violet-650-anti-human-hla-dr-antibody-8875
Anti-PD-1 https://www.biolegend.com/en-us/products/apc-anti-human-cd279-pd-1-antibody-4413
Anti-PD-L1 https://www.biolegend.com/en-us/products/apc-anti-human-cd274-b7-h1-pd-l1-antibody-16138
Anti-CD69 https://www.bdbiosciences.com/en-ca/products/reagents/flow-cytometry-reagents/research-reagents/single-color-antibodies-ruo/pe-mouse-anti-human-cd69.557050
Anti-mIgG1 https://www.bdbiosciences.com/en-ca/products/reagents/flow-cytometry-reagents/research-reagents/flow-cytometry-controls-and-lysates/pe-mouse-igg1-isotype-control.555749
Anti-mIgG1 https://www.bdbiosciences.com/en-ca/products/reagents/flow-cytometry-reagents/research-reagents/flow-cytometry-controls-and-lysates/apc-mouse-igg1-isotype-control.550854
Anti-Cytokeratin https://www.miltenyibiotec.com/LU-en/products/cytokeratin-antibody-anti-human-reafinity-rea831.html#pe:100-tests-in-200-ul
Anti-CD2 https://www.miltenyibiotec.com/LU-en/products/cd2-antibody-anti-human-reafinity-rea1130.html#pe:200-ul
Anti-CD279 https://www.miltenyibiotec.com/LU-en/products/cd279-pd1-antibody-anti-human-reafinity-rea1165.html#pe:100-tests-in-200-ul
Anti-CD3 https://www.miltenyibiotec.com/LU-en/products/cd3-antibody-anti-human-reafinity-rea1151.html#fitc:200-ul

Eukaryotic cell lines

Policy information about cell lines

Cell line source(s)	A549, NCI-H460, NCI-H23, NCI-H226, NCI-H322, NCI-H522, HOP-62 and HOP-92 were obtained from the American Type Culture Collection (ATCC).
Authentication	Authentication numbers are provided in the manuscript.
Mycoplasma contamination	Cell lines were tested negative for mycoplasma contamination.
Commonly misidentified lines (See ICLAC register)	No cell line used in this paper is listed in the ICLAC register.

Human research participants

Policy information about studies involving human research participants

Population characteristics	The inclusion criteria in this study were defined as follows: male or female, age > 18 years and histologically confirmed NSCLC. Summary statistics are provided in Supplementary Fig. 4 and Supplementary Table 1.
Recruitment	For determination of pPD-L1, patients with NSCLC treated at the University-Hospital Department of Medical Oncology and Hematology and the Department of Internal Medicine VIII, University Hospital Tuebingen, Germany were prospectively recruited by their treating physicians on predetermined in- and exclusion criteria. Recruiting physicians were not involved in sample preparation and consecutive analysis. Clincial data analysis were performed from operators not involved in the recruiting process.
Ethics oversight	Written informed consent was given in all cases. This study was approved by IRB (ethics committee of the Faculty of Medicine of the Eberhard Karls University Tuebingen) and of the University Hospital Tuebingen and was conducted in accordance with the Declaration of Helsinki; reference number 456/BO2.

Note that full information on the approval of the study protocol must also be provided in the manuscript.

Flow Cytometry

Plots

Confirm that:

- [x] The axis labels state the marker and fluorochrome used (e.g. CD4-FITC).
- [x] The axis scales are clearly visible. Include numbers along axes only for bottom left plot of group (a 'group' is an analysis of identical markers).
- [x] All plots are contour plots with outliers or pseudocolor plots.
- [x] A numerical value for number of cells or percentage (with statistics) is provided.

Methodology

Sample preparation	Depending on the respective staining, cells and/or platelets were fixed in 2% PFA in PBS (pH 7.4) for 10 min at -20°C followed by two wash steps with PBS (3% FBS). For T cell activation, cells were incubated with, 10 µg ml-1 brefeldin A and 1:500 dilution of GolgiStop (CD) for 12-16h. Staining was performed using fluorescence-conjugates or specific mAb and their controls.

Instrument	FACS Canto II cytometer (BD)
Software	FlowJo software version 10.0.8 (BD)
Cell population abundance	Cells have not been enriched or sorted prior in vitro stimulation. Culturing peripheral blood mononuclear cells were co-incubated for 12 days in the presence of recombinant IL-2 for T cell enrichment. For generation on NY-ESO-1 specific T cells, cells were stimulated using pools of NY-ESO-1 overlapping peptides and cultured in RPMI 1640 containing AB-serum and L-glutamin in the presence of recombinant IL-1 and IL-7 for 7-14 days. NY-ESO-1 specific, IFNy+ T cells were enriched after re-stimulation with NY-ESO-1 peptide pool for 6 h using CliniMACS® technique.
Gating strategy	Platelets were selected in the FSC/SSC gate and identified as subcellular CD41, CD61 and/or CD62P expressing particles. Lymphocytes were selected in the FSC/SSC gate. Single cells were selected using FSC-H/FSC-A. Viable cells were identified using Aqua live/dead staining. Cells expressing either CD4 or CD8 were analyzed for production of indicated cytokines and expression markers. Tumor cell lines were selected in the FSC/SSC gate. Isotype controls were used to confirm the specificity of the staining.

☒ Tick this box to confirm that a figure exemplifying the gating strategy is provided in the Supplementary Information.
