# Supplementary material for: Platelet PD-L1 reflects collective intratumoral PD-L1 expression and predicts immunotherapy response in non-small cell lung cancer
Source: Nat Commun. 2021 Dec 1;12:7005. doi: 10.1038/s41467-021-27303-7 (PMC8636618; doi:10.1038/s41467-021-27303-7)
Supplement: Supplementary file 3 — Description of Additional Supplementary Files [file 41467_2021_27303_MOESM3_ESM.pdf]

Description of Additional Supplementary Files:

File Name: Supplementary Movie 1

Description: Tracking platelet tumor cell interaction using live cell imaging.

File Name: Supplementary Movie 2

Description: Tracking platelet tumor cell interaction using live cell imaging (close-up).
